# Changes in Gastric Corpus Microbiota With Age and After *Helicobacter pylori* Eradication: A Long-Term Follow-Up Study

**DOI:** 10.3389/fmicb.2020.621879

**Published:** 2021-02-09

**Authors:** Cheol Min Shin, Nayoung Kim, Ji Hyun Park, Dong Ho Lee

**Affiliations:** ^1^Department of Internal Medicine, Seoul National University Bundang Hospital, Seongnam-si, South Korea; ^2^Department of Internal Medicine and Liver Research Institute, Seoul National University College of Medicine, Seoul, South Korea

**Keywords:** *Helicobacter pylori*, gastric microbiota, eradication, intestinal metaplasia, atrophic gastritis

## Abstract

*Helicobacter pylori* infection changes gastric microbiota profiles. However, it is not clear whether *H. pylori* eradication can restore the healthy gastric microbiota. Moreover, there has been no study regarding the changes in gastric microbiota with aging. The objective of this study was to investigate the changes in gastric corpus microbiota with age and following *H. pylori* eradication. Changes in corpus mucosa-associated microbiota were evaluated in 43 individuals with endoscopic follow-up > 1 year, including 8 *H. pylori*-uninfected and 15 *H. pylori*-infected subjects with no atrophy/metaplasia by histology and pepsinogen I/II ratio > 4.0; 17 *H. pylori*-infected subjects with atrophy/metaplasia and pepsinogen I/II ratio < 2.5; and 3 subjects with atrophy/metaplasia, no evidence of active *H. pylori* infection, negative for anti-*H. pylori* immunoglobulin G (IgG) antibody testing, and no previous history of *H. pylori* eradication. Successful *H. pylori* eradication was achieved in 21 patients. The gastric microbiota was characterized using an Illumina MiSeq platform targeting 16S ribosomal DNA (rDNA). The mean follow-up duration was 57.4 months (range, 12–145 months), and median follow-up visit was 1 (range, 1–3). Relative abundance of Lactobacillales and *Streptococcus* was increased with atrophy/metaplasia. In *H. pylori*-uninfected subjects (*n* = 8), an increase in Proteobacteria (*Enhydrobacter*, Comamonadaceae, *Sphingobium*); a decrease in Firmicutes (*Streptococcus*, *Veillonella*), Fusobacteria (*Fusobacterium*), Nocardioidaceae, *Rothia*, and Prevotella; and a decrease in microbial diversity were observed during the follow-up (*p* trend < 0.05). In 10 of 21 subjects (47.6%), *H. pylori* eradication induced restoration of microbial diversity; however, a predominance of *Acinetobacter* with a decrease in microbial diversity occurred in 11 subjects (52.3%). The presence of atrophy/metaplasia at baseline and higher neutrophil infiltration in the corpus were associated with the restoration of gastric microbiota after successful eradication, whereas a higher relative abundance of *Acinetobacter* at baseline was associated with the predominance of *Acinetobacter* after *H. pylori* eradication (*p* < 0.05). To conclude, in *H. pylori-*uninfected stomach, relative abundance of Proteobacteria increases, relative abundance of Firmicutes and Fusobacteria decreases, and microbial diversity decreases with aging. *H. pylori* eradication does not always restore gastric microbiota; in some individuals, gastric colonization by *Acinetobacter* species occurs after anti-*Helicobacter* treatment.

## Introduction

The stomach is a hostile environment for most bacteria due to high acidity and various antimicrobial chemicals and enzymes. *Helicobacter pylori* used to be considered the only exception. *H. pylori* infection is associated with peptic ulcer diseases, low-grade gastric mucosa-associated lymphoma, and gastric cancer (GC) (Cover and Blaser, 2009). To prevent these *H. pylori*-related diseases, eradication of *H. pylori* is recommended. *H. pylori* infection plays a key role in the initial steps of gastric carcinogenesis by causing chronic active inflammation and progressive damage to the gastric epithelium (Correa, 1992). However, studies on *H. pylori* eradication have shown that successful eradication alone does not completely prevent the risk of gastric cancer (Correa, 1992; Choi et al., 2018; Li et al., 2019), and it may increase the risk for *H. pylori*-negative GC (Lee et al., 2019). Therefore, it has been suggested that factors other than *H. pylori* contribute to the development of gastric cancer.

It has been hypothesized that chronic *H. pylori* infection leads to a decrease in gastric acid secretion, which allows successful colonization by new bacteria species that may contribute to gastric carcinogenesis by causing persistent inflammation and conversion of nitrates into N-nitrosamines (Bik et al., 2006; Andersson et al., 2008). Reduction in gastric acidity by antisecretory agents may result in substantial intragastric bacterial overgrowth, increased counts of nitrate-reducing bacteria, and increased nitrite and N-nitrosamine levels (Stockbruegger, 1985; Leach et al., 1987). This has been confirmed by studies using the hypergastrinemic insulin-gastrin (INS-GAS) transgenic mouse model, which showed that *H. pylori*-induced gastric carcinogenesis was facilitated by the presence of a complex gastric microbiota, with the mice developing more tumors than germ-free mice infected with *H. pylori* only (Lofgren et al., 2011; Lertpiriyapong et al., 2014).

Recently, next-generation sequencing (NGS) has allowed the investigation of the human gastric microbiota in gastric fluid and biopsy samples, which showed that there are diverse bacterial taxa in the stomach dominated by five phyla, including Actinobacteria, Bacteroidetes, Firmicutes, Fusobacteria, and Proteobacteria (Bik et al., 2006; Andersson et al., 2008). *H. pylori* infection substantially alters gastric microbiota profiles, but previous results on this issue have been inconsistent (Aviles-Jimenez et al., 2014; Lertpiriyapong et al., 2014; Jo et al., 2016; Ferreira et al., 2018). A pilot study has reported that *H. pylori* colonization altered the gastric microbiota and reduced microbial diversity, which could be restored by *H. pylori* eradication (Li et al., 2017). However, there are concerns regarding dysbiosis caused by antibiotic therapy.

Previously, we have shown that the lower part of rat gastric mucosa was replaced by connective tissue and characterized by the accumulation of oxidative products with aging (Kang et al., 2010). In addition, rat aging has been associated with significantly decreased basal and stimulated gastric acid levels as well as with decreased expression of messenger RNA (mRNA) and protein of H^+^-K^+^-ATPase (Jo et al., 2013). Thus, gastric microbiota may change with age. However, there has been no study regarding the changes in gastric microbiota with aging in *H. pylori*-uninfected individuals. Additionally, the microbiota profiles are different between the gastric antrum and corpus. Recently, we have shown that mucosa-associated microbiota in the corpus, not in the antrum, could be useful in identifying the role of *non-H. pylori* bacteria in gastric carcinogenesis (Jo et al., 2016; Sohn et al., 2017). Therefore, we investigated the time course of changes in gastric corpus microbiota with age and following *H. pylori* eradication.

## Materials and Methods

### Study Subjects

Changes in gastric corpus mucosa-associated microbiota were evaluated in 43 individuals without significant gastroduodenal disease (Supplementary Figure 1). All subjects received initial endoscopy at baseline, and at least one endoscopy was performed > 1 year after the enrollment. None of them were identified as using proton pump inhibitors (PPIs), histamine-2 receptor antagonists, or non-steroidal anti-inflammatory drugs at baseline and follow-up; they had no history of antibiotic use for at least 4 weeks before sample collection. The study participants were classified into four groups: *H. pylori*-uninfected subjects without evidence of atrophic gastritis and intestinal metaplasia by histology, pepsinogen I/II ratio ≥ 4.0, and no history of *H. pylori* eradication (group 1, *n* = 8); *H. pylori*-infected patients without mucosal atrophy and metaplasia by histology with pepsinogen I/II ratio > 4.0 (group 2, *n* = 15); *H. pylori*-infected patients with atrophic gastritis and/or intestinal metaplasia by histology and pepsinogen I/II ratio < 2.5 (group 3, *n* = 17); and the subjects with atrophic gastritis and/or intestinal metaplasia, no evidence of current *H. pylori* infection, anti-*H. pylori* immunoglobulin G (IgG) antibody negative, and no previous history of *H. pylori* eradication (group 4, *n* = 3).

Initially, 27 of 32 *H. pylori-*infected patients (groups 2 and 3) underwent *H. pylori* eradication therapy; 23 of them received the standard triple therapy (a standard dose of PPI + 1,000 mg amoxicillin + 500 mg clarithromycin twice a day for 7–14 days), 2 received a bismuth-based quadruple therapy (two standard doses of PPI, three doses of 500 mg metronidazole, four doses 120 mg bismuth for 7 days), and 2 received sequential therapy (5 days of treatment with PPI and amoxicillin, followed by another 5 days of treatment with PPI, clarithromycin, and metronidazole). Among the 27 patients, *H. pylori* was successfully eradicated without reinfection in 15 patients. Reinfection was defined as positive for either rapid urease test or *H. pylori* histology at follow-up endoscopy. Among the 12 patients whose *H. pylori* infection was not treated or failed to be eradicated, 6 showed eradication during follow-up: 3 patients were treated with bismuth-based quadruple therapy, 1 with standard triple therapy, and 2 with moxifloxacin-based triple therapy. Finally, *H. pylori* was eradicated in 21 patients. A flow chart of the study is presented in Supplementary Figure 1.

This study was approved by the Institutional Review Board of Seoul National University Bundang Hospital (B-1903/529-302). All study procedures involving human participants were in accordance with the 1964 Declaration of Helsinki and its later amendments or comparable ethical standards. All study participants signed a consent form before enrolling in the study.

### *H. pylori* Testing and Histology

At each endoscopy, 10 biopsy specimens were obtained for histological analysis, *Campylobacter*-like organism (CLO) test, and culture to determine the presence of a current *H. pylori* infection. This methodology has been presented previously (Shin et al., 2013). In brief, two biopsy specimens from the greater curvature side of the antrum and two from the body were fixed in formalin to assess the presence of *H. pylori* by modified staining and the degree of inflammatory cell infiltration, atrophy, and intestinal metaplasia (all by hematoxylin and eosin staining). These histological features of the gastric mucosa were recorded using the updated Sydney scoring system (0, none; 1, mild; 2, moderate; and 3, marked) (Dixon et al., 1996). One specimen from each of the lesser curvature of the antrum and the body was used for rapid urease testing (CLOtest, Delta West, Bentley, Australia), and two specimens, one from the antrum and one from the body, were used for culture, and the organisms present were identified as *H. pylori* by Gram staining, colony morphology, and positive oxidase, catalase, and urease reactions. The remaining biopsy specimens and gastric cancer tissues were immediately frozen at -70°C until DNA extraction.

### *H. pylori* Serology and the Evaluation of Gastric Atrophy by Serum Pepsinogen Tests

Fasting serum samples were collected from the study subjects at baseline. For *H. pylori* serology testing, specific IgG for *H. pylori* was identified by an enzyme-linked immunosorbent assay in each subject’s serum (Genedia *H. pylori* ELISA; Green Cross Medical Science Corp., Eumsung, Korea); Korean strain was used as antigen in this *H. pylori* antibody test (Kim et al., 2014). In addition, serum concentrations of pepsinogen (PG) I and II were measured using a Latex-enhanced turbidimetric immunoassay (Shima Laboratories, Tokyo, Japan). In this study, no atrophy was defined as PG I > 70 and PG I/II ratio > 4.0. Atrophy was defined as a PG I/II ratio < 2.5.

### DNA Preparation

Genomic DNA was extracted directly from non-cancerous corporal biopsy specimens (Shin et al., 2013). Briefly, specimens were homogenized in proteinase K solution [20 mmol/L Tris–HCl (pH 8.0), 10 mmol/L ethylenediaminetetraacetic acid, 0.5% sodium dodecyl sulfate, and 10 mg/ml proteinase K] using a sterile micropestle and then incubated for 3 h at 52°C. DNA was isolated from homogenates using phenol/chloroform extraction and ethanol precipitation.

### PCR Amplification and Illumina Sequencing

PCR amplification was performed using primers targeting from V3 to V4 regions of the 16S rRNA gene of the extracted DNA. For bacterial amplification, the primers were as follows: 341F (5′-TCGTCGGCAGCGTC-AGATGTGTATAAGAGACAG-CCTA CGGGNGGCWGCAG-3′) and 805R (5′-GTCTCGTGGGCT CGGAGATGTGTATAAGAGACAGGACTACHVGGGTATCTA ATCC-3′). The amplifications were carried out under the following conditions: initial denaturation at 95°C for 3 min, followed by 25 cycles of denaturation at 95°C for 30 s, primer annealing at 55°C for 30 s, and extension at 72°C for 30 s, with a final elongation at 72°C for 5 min. Then, secondary amplification for attaching the Illumina NexTera barcode was performed using the i5 forward primer (5′-AATGATACGGCGACCACCGAGATCTACAC-XXXXXXXX-TCGTCGGCAGCGTC-3′; X indicates the barcode region) and the i7 reverse primer (5′-CAAGCAGA AGACGGCATACGAGATXXXXXXXX-AGTCTCGTGGGCTC GG-3′). The conditions of the secondary amplification were identical to those reported above with the exception that the amplification cycle was set to 8. The PCR product was confirmed by 1% agarose gel electrophoresis and visualized using the Gel Doc system (BioRad, Hercules, CA, United States). The amplified products were purified using the QIAquick PCR Purification Kit (Qiagen, Valencia, CA, United States). Equal concentrations of the purified products were pooled together, and the short fragments (non-target products) were removed using an Ampure beads kit (Agencourt Bioscience, MA, United States). The quality and product size were assessed on a Bioanalyzer 2100 (Agilent, Palo Alto, CA, United States) using a DNA 7500 chip. Mixed amplicons were pooled, and the sequencing was carried out at Chunlab, Inc. (Seoul, Korea) on an Illumina MiSeq Sequencing system (Illumina, United States) according to the manufacturer’s instructions.

### MiSeq Pipeline Method

Processing raw reads started with quality check and filtering of low-quality (< Q25) reads using Trimmomatic 0.32 (Bolger et al., 2014). After quality control (QC) pass, paired-end sequence data were emerged together using PANDAseq (Masella et al., 2012). Primers were then trimmed using ChunLab’s in-house program at a similarity cutoff of 0.8. Non-specific amplicons that did not encode 16S rRNA were detected using the hmmsearch program of HMMER (Eddy, 2011) with 16S rRNA profiles. Sequences were denoised using DUDE-Seq (Lee et al., 2017), and non-redundant reads were extracted by UCLUST clustering (Edgar, 2010). The EzBioCloud database was used for taxonomic assignment using USEARCH (8.1.1861_i86linux32) (Edgar, 2010) followed by more precise pairwise alignment (Myers and Miller, 1988). UCHIME (Edgar et al., 2011) and the non-chimeric 16S rRNA database from EzBioCloud were used to detect chimera on reads that showed < 97% best hit similarity rate. Sequence data were then clustered using CD-HIT (Fu et al., 2012) and UCLUST (Edgar, 2010). The α-diversity indices and rarefaction curves were estimated using an in-house code.

### Nitrosating Bacteria/Urease-Producing Bacteria

The rRNA sequence data were intensively analyzed with a focus on bacteria that have the ability to nitrosate or reduce nitrate such as *Clostridium*, *Veillonella*, *Haemophilus*, *Staphylococcus*, and *Neisseria*, which were identified in previous studies. In addition, urease-producing bacteria other than *H. pylori* were also identified and classified during the analysis (Supplementary Table 1 lists the specific species included in the analysis).

### Statistical Analysis

In this study, operational taxonomic units (OTUs) were collapsed by shared taxonomy at all taxonomic levels from phylum to genus. Taxa with an average relative abundance of < 1% were discarded. Alpha-diversity indices (i.e., observed species, phylogenetic whole tree, and Shannon diversity index) were estimated and tested for significant differences between the groups using ANOVA tests. Beta-diversity distances (unweighted/weighted UniFrac and Bray–Curtis distances) were calculated on rarefied taxa tables. Permutational multivariate analysis of variance (PERMANOVA) and homogeneity of dispersion tests were performed using the “adonis” function in the “vegan” package in R (Dixon, 2003). Linear discriminant analysis effect size (LEfSe) was used to identify the biomarkers significantly enriched in each group (Segata et al., 2011). Wilcoxon rank-sum test (Mann–Whitney test) was performed to infer the significant difference in diversity and microbial compositions between the groups. The paired Wilcoxon rank-sum test was used to identify the significant changes in taxa in pre- and posteradication samples. To model the time course of gastric microbiota changes in *H. pylori*-uninfected individuals (group 1), a linear mixed model was applied, as it incorporates a generic correlation structure of longitudinal data by considering within- and between-subject variations (Shin et al., 2013). False discovery rate (FDR)-corrected *q*-values were calculated with a significance threshold of 5%.

## Results

### Characteristics of the Study Participants

Table 1 shows the baseline characteristics of the study subjects. The mean follow-up duration was 57.4 months (range, 12–145 months), and the median follow-up visits were 1 (range, 1–3). The bacterial composition of all study subjects (*n* = 43) at the phylum level is presented in Supplementary Figure 2.

**TABLE 1:** Baseline characteristics of the study subjects (*N* = 43).

	Group 1 *Hp* (−), AG (−)	Group 2 *Hp* (+), AG (−)	Group 3 *Hp* (+), AG(+)	Group 4 *Hp* (−), AG(+)	*p*-Value

	*n* = 8	*n* = 15	*n* = 17	*n* = 3	
Male, n (%)	3 (37.5)	5 (33.3)	3 (17.6)	0 (0.0)	0.446
Age, years	49.6 ± 9.8	50.9 ± 11.7	58.3 ± 10.4	62.0 ± 10.4	0.092
FU duration, m, median (range)	93 (12–129)	44 (13–102)	81 (14–132)	56 (40–99)	NS
FU times, median (range)	2 (1–3)	1 (1–3)	2 (1–4)	2 (1–3)	NS
Current smoker (*n* = 41), n (%)	2 (25.0)	1 (6.7)	0 (0.0)	0 (0.0)	0.163
Drinker, n (%)	3 (37.5)	4 (26.7)	1 (5.9)	0 (0.0)	0.194
Education					**0.034**
Elementary–middle–high	7 (87.5)	4 (33.3)	11 (68.8)	3 (100)	
University	1 (12.5)	8 (66.7)	5 (31.3)	0 (0.0)	
**Histology (updated Sydney classification)**					
***Hp***					
Antrum	0.0 ± 0.0	1.6 ± 1.0	1.4 ± 0.9	0.0 ± 0.0	** < 0.001**
Corpus	0.0 ± 0.0	1.9 ± 0.9	2.1 ± 0.7	0.0 ± 0.0	** < 0.001**
**Neutrophil infiltration**					
Antrum	0.1 ± 0.4	1.9 ± 0.6	1.6 ± 0.9	0.0 ± 0.0	** < 0.001**
Corpus	0.1 ± 0.4	1.4 ± 0.9	2.1 ± 0.3	0.3 ± 0.6	** < 0.001**
**Monocyte infiltration**					
Antrum	0.0 ± 0.0	0.1 ± 0.3	1.3 ± 0.5	0.0 ± 0.0	** < 0.001**
Corpus	0.0 ± 0.0	0.0 ± 0.0	1.0 ± 0.7	2.3 ± 0.6	** < 0.001**
**AG**					
Antrum	0.0 ± 0.0	0.1 ± 0.3	1.3 ± 0.5	0.0 ± 0.0	** < 0.001**
Corpus	0.0 ± 0.0	0.0 ± 0.0	1.0 ± 0.7	2.3 ± 0.6	** < 0.001**
**IM**					
Antrum	0.0 ± 0.0	0.1 ± 0.3	1.2 ± 0.8	1.0 ± 0.0	** < 0.001**
Corpus	0.0 ± 0.0	0.0 ± 0.0	0.6 ± 0.8	1.7 ± 0.6	** < 0.001**
PGI	50.6 ± 15.2	98.5 ± 91.6	45.7 ± 14.3	10.6 ± 10.3	**0.026**
PG II	9.0 ± 2.0	19.4 ± 10.8	22.2 ± 8.1	13.4 ± 6.6	**0.006**
PG I/II ratio	5.6 ± 0.8	5.5 ± 3.7	2.2 ± 0.8	0.7 ± 0.4	** < 0.001**

*p-Values were calculated using chi-square test or Student’s t-test. Bold style indicates statistical significance. Hp, Helicobacter pylori; AG, atrophic gastritis; IM, intestinal metaplasia; PG, pepsinogen; FU, follow-up; NS, non-significant.*

### Microbial Diversity, Bacterial Composition, and Composition of Nitrosating/Urease-Producing Bacteria According to *H. pylori* Infection and Presence of Mucosal Atrophy or Intestinal Metaplasia

**FIGURE 1:**
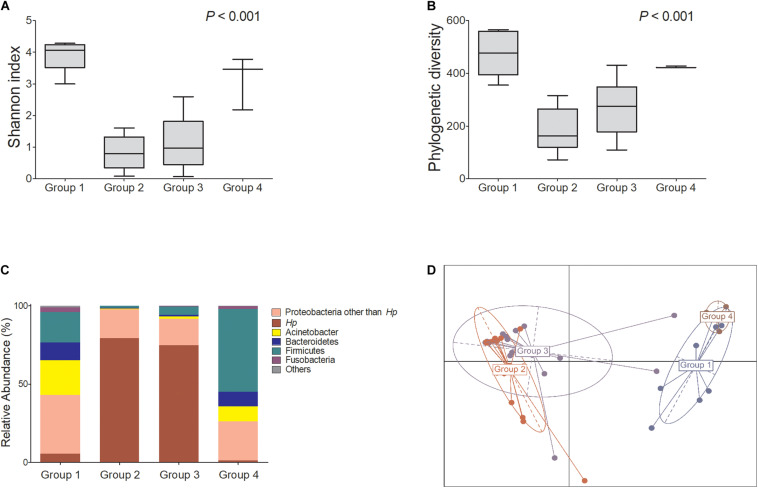
Microbiota composition at gastric corpus mucosae according to *Helicobacter pylori* infection and the presence of atrophy/metaplasia. *H. pylori* infection decreased the microbial diversity at corpus (**A**,**B** groups 2 and 3). Compared with group 1 (*H. pylori*-uninfected individuals), *H. pylori* predominates in the gastric corpus microbiota in groups 2 and 3 (*H. pylori*-infected subjects), and relative abundance of Firmicutes was increased in group 4 (**C** *H. pylori*-negative subjects with atrophy/metaplasia). **(D)** Principal coordinates analysis showed that the study subjects can be clearly discriminated by *H. pylori* infection status (groups 1 and 4 vs. groups 2 and 3) but not by the presence or absence of atrophy/metaplasia. The four groups were significantly different (Bray–Curtis, unweighted, PERMANOVA *p* < 0.001). Group 1: *H. pylori*-uninfected subjects without evidence of atrophic gastritis and intestinal metaplasia by histology, pepsinogen I/II ratio ≥ 4.0, and no history of *H. pylori* eradication (*n* = 8); group 2: *H. pylori*-infected patients without mucosal atrophy and metaplasia by histology with pepsinogen I/II ratio > 4.0 (*n* = 15); group 3: *H. pylori*-infected patients with atrophic gastritis and/or intestinal metaplasia by histology and pepsinogen I/II ratio < 2.5 (*n* = 17); group 4: the patients with atrophy/metaplasia, no evidence of active *H. pylori* infection, negative for anti-*H. pylori* IgG antibody test, and no previous history of *H. pylori* eradication (*n* = 3). *Hp*, *Helicobacter pylori*.

Figure 1 shows the microbial diversity, composition of bacterial taxa, and nitrosating and urease-producing bacteria according to the *H. pylori* infection status and the presence of mucosal atrophy and/or intestinal metaplasia. *H. pylori* infection significantly decreased the microbial diversity indices (Figure 1A: Shannon index; Figure 1B: phylogenetic diversity). The principal coordinates analysis (PCoA) plot of all study participants (*n* = 43, Bray–Curtis, unweighted) is presented in Figure 1D, which shows that the four groups were significantly different (PERMANOVA, *p* < 0.001). In detail, *H. pylori*-positive groups (groups 2 and 3) and *H. pylori*-negative groups (groups 1 and 4) did not overlap with each other, but the four groups were not distinguished from each other by the presence or absence of atrophic gastritis or intestinal metaplasia. In *H. pylori*-negative subjects with atrophy/metaplasia (group 4), the relative abundance of nitrosating bacteria other than *H. pylori* was increased (Supplementary Figure 3A). In group 4, the relative abundance of urease-producing bacteria other than *H. pylori* was significantly increased compared with that in other groups (Supplementary Figure 3B). In *H. pylori*-positive subjects with atrophy/metaplasia (group 3), the relative abundance of urease-producing bacteria other than *H. pylori* was higher than in *H. pylori*-positive subjects without atrophy or metaplasia (group 2).

### Gastric Corpus Microbiota Changes Caused by *H. pylori* Infection in Subjects Without Mucosal Atrophy and Metaplasia

LEfSe analysis was performed to identify taxa that were significantly enriched in each group. Among subjects without atrophy or metaplasia (groups 1 and 2, *n* = 23), *H. pylori* infection significantly changed mucosa-associated gastric microbiota in the corpus (decrease in Actinobacteria, Sphingomonadales, *Prevotella*, and *Veillonella* abundance; FDR *q* < 0.05 by LEfSe analysis of group 1 vs. group 2), which resulted in a decrease in microbial diversity (*p* < 0.01, Shannon and phylogenetic diversity indices, Supplementary Figure 4).

### Gastric Corpus Microbiota Changes Due to Mucosal Atrophy and Intestinal Metaplasia

**FIGURE 2:**
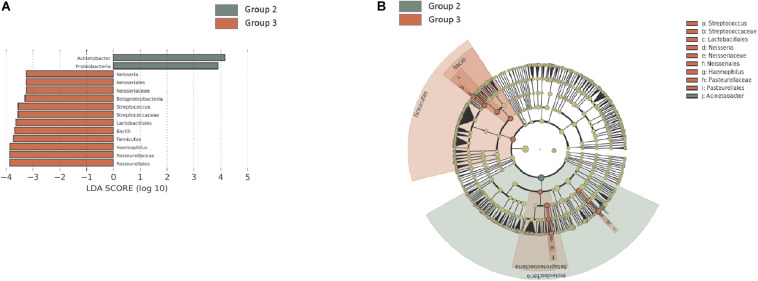
Summary of the linear discriminant analysis (LEfSe) comparing group 2 with group 3. **(A)** LDA scores and **(B)** cladogram show significantly different taxa [*p* < 0.05 and Log_10_ (LDA score) > 3.0]. Among *H. pylori*-positive patients (*n* = 32), relative abundance of Firmicutes, especially *Streptococcus* and Lactobacillales, was significantly increased in group 3 compared with that in group 2. Group 2: *H. pylori*-infected patients without mucosal atrophy and metaplasia by histology with pepsinogen I/II ratio > 4.0 (*n* = 15); group 3: *H. pylori*-infected patients with atrophic gastritis and/or intestinal metaplasia by histology and pepsinogen I/II ratio < 2.5 (*n* = 17).

Among *H. pylori*-positive patients (groups 2 and 3, *n* = 32), the relative abundance of Firmicutes, especially *Streptococcus* and Lactobacillales, was significantly higher in group 3 compared with that in group 2. The relative abundance of Proteobacteria, especially *Acinetobacter* spp., was increased in group 3, whereas the abundance of Neisseria was increased in group 2 (Figures 2A,B).

Among *H. pylori*-negative subjects (groups 1 and 4, *n* = 11), the relative abundance of Firmicutes, including *Streptococcus*, *Parvimonas*, and Lactobacillales, was increased in subjects with atrophy/metaplasia (group 4) compared with subjects without atrophy/metaplasia (group 1, Supplementary Figures 5A,B). In contrast, the relative abundance of alphaproteobacteria (*Methylobacterium* and *Paracoccus*), Chloroflexi (Thermomicrobia), Corynebacteriales, Microbacteriaceae, Propionibacteriaceae (*Cutibacterium*), and Burkholderiales (*Massilia*, *Diaphorobacter*, and *Comamonadaceae*) was decreased in group 4.

### Long-Term Follow-Up of *H. pylori*-Negative Individuals Without Atrophic Gastritis and Intestinal Metaplasia

**FIGURE 3:**
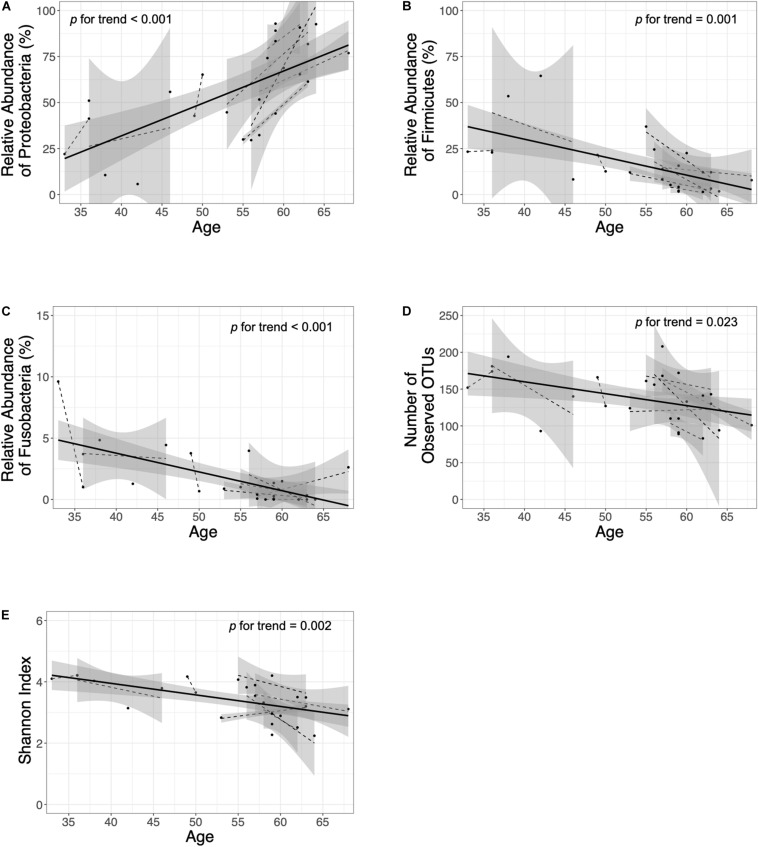
Follow-up of the *H. pylori*-negative/non-atrophy subjects (*n* = 8). At phylum level, an increase in **(A)** Proteobacteria abundance and a decrease in **(B)** Firmicutes and **(C)** Fusobacteria abundance were observed during the follow-up. In addition, a decrease in α-diversity indices (number of observed OTUs and Shannon index are presented in panel **D** and **E**, respectively) were observed. OTUs, operational taxonomy units.

In the *H. pylori*-negative non-atrophy group (group 1, *n* = 8), gastric corpus microbiota consisted of Proteobacteria (38.5%), Actinobacteria (23.3%), Firmicutes (21.2%), and Bacteroidetes (12.3%, Figure 1C). No individuals in group 1 developed atrophic gastritis or intestinal metaplasia during the follow-up period of up to 10 years; none of them were infected with *H. pylori* during the follow-up (*H. pylori* infection was identified as positive for either rapid urease test or *H. pylori* histology). Interestingly, at phylum level, an increase in Proteobacteria abundance was observed during the endoscopic follow-up (*p-*value for trend = 0.00002, Figure 3A). In contrast, the relative abundance of Firmicutes and Fusobacteria showed a significant decrease with aging (*p* for trend = 0.001 and < 0.001, respectively, Figures 3B,C). At family level, an increase in Moraxellaceae and Comamonadaceae abundance and a decrease in Streptococcaceae, Prevotellaceae, Veillonellaceae, Nocardioidaceae, Fusobacteriaceae, Actinomycetaceae abundance were observed during the follow-up (FDR *q*-value < 0.05, Supplementary Figure 6A). At genus level, the relative abundance of *Enhydrobacter*, *Sphingobium*, and *Chryseobacterium* was increased and that of *Streptococcus*, *Prevotella*, *Veillonella*, *Rothia*, and *Fusobacterium* was decreased (FDR *q*-value < 0.05, Supplementary Figure 6B). Taxonomic associations with aging are summarized in Supplementary Table 2. In addition, a decrease in microbial diversity was noted during the follow-up periods (*p* for trend = 0.0231 and 0.0018 by the observed operational taxonomic unit counts and Shannon index, respectively, Figures 3D,E). The changes in bacterial composition at the phylum level in eight individuals in group 1 are presented in Supplementary Figure 7.

### Follow-Up of Gastric Corpus Mucosa-Associated Microbiota After *H. pylori* Eradication

**FIGURE 4:**
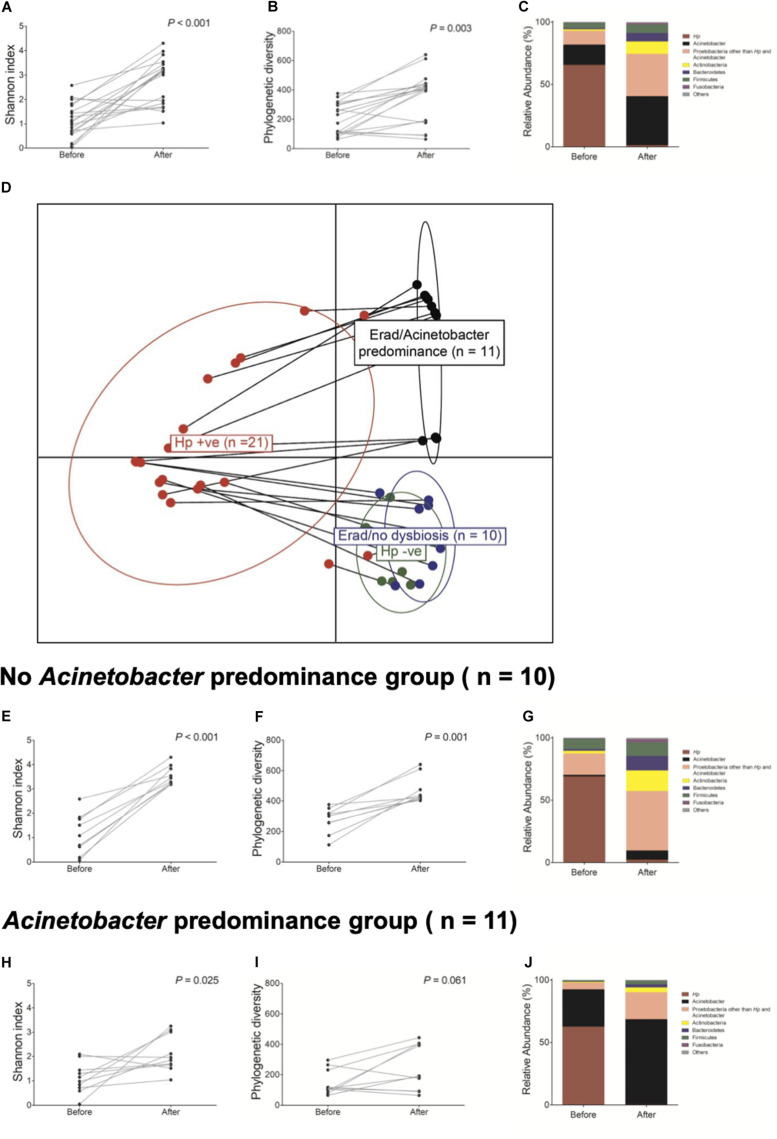
Changes in gastric microbiota after *H. pylori* eradication. *H. pylori* eradication led to an **(A,B)** increase in microbial diversity and a **(C)** restoration of gastric microbiota. After *H. pylori* eradication, two district clusters can be identified (**D** eradicated without *Acinetobacter* predominance vs. eradicated with *Acinetobacter* predominance). **(E–G)** In the eradicated without *Acinetobacter* predominance group (*n* = 10), eradication of *H. pylori* led to a restoration of diverse gastric microbiota composition. **(H–J)** However, in the eradicated with *Acinetobacter* predominance group (*n* = 11), severe dysbiosis with an increase in *Acinetobacter* species abundance and a decrease in microbial diversity was observed.

Next, we investigated whether *H. pylori* eradication could restore gastric microbiota in the corpus. The changes in bacterial composition at the phylum level in these 21 patients are presented in Supplementary Figure 8. Overall, *H. pylori* eradication increased the microbial diversity indices (Figures 4A,B). However, the consequences of *H. pylori* eradication can be clustered into two groups [eradicated without *Acinetobacter* predominance group (*n* = 10) vs. eradicated with *Acinetobacter* predominance group (*n* = 11), Figure 4D]. In 10 of 21 (47.6%) subjects in the “eradicated without *Acinetobacter* predominance” group, eradication of *H. pylori* led to a restoration of the diverse gastric microbiota composition (Figures 4E–G). However, in 11 (52.4%) patients in the “eradicated with *Acinetobacter* predominance” group, severe dysbiosis due to an increase in abundance of the *Acinetobacter* genus with a decrease in microbial diversity was observed (Figures 4H–J).

Table 2 shows predictable factors of dysbiosis after successful *H. pylori* eradication. Among the clinical variables, the presence of atrophy/metaplasia at baseline (*p* = 0.001), higher neutrophil infiltration at corpus (*p* = 0.034), and lower PG I/II ratio (*p* = 0.022) were significantly associated with the restoration of gastric microbiota after successful anti-*Helicobacter* treatment. In addition, higher relative abundance of *Acinetobacter* was significantly associated with *Acinetobacter* predominance after *H. pylori* eradication (*p* = 0.005).

**TABLE 2:** Comparison of clinical variables, microbiota composition, and histology between the no dysbiosis group (*n* = 10) and the dysbiosis group (*n* = 11) after successful *H. pylori* eradication.

	No dysbiosis	Dysbiosis	*p*-Value

	*n* = 10	*n* = 11	
Male, n (%)	4 (40.0)	4 (36.4)	0.864
Age, years	58.3 ± 10.4	62.0 ± 10.4	0.092
Current smoker (*n* = 20), n (%)	0 (0.0)	1 (10.0)	1.000
Drinker, n (%)	2 (20.0)	2 (18.2)	1.000
AG or IM*	9 (90.0)	4 (36.4)	**0.011**
Relative abundance of *Acinetobacter* genus at baseline	1.3 ± 2.1	29.8 ± 26.5	**0.005**
**Histology at baseline (updated Sydney classification)**			
***H. pylori***			
Antrum	1.5 ± 1.1	1.5 ± 0.7	0.909
Corpus	1.8 ± 0.8	1.8 ± 1.0	0.963
**Neutrophil infiltration**			
Antrum	1.5 ± 1.1	2.1 ± 0.3	0.125
Corpus	2.2 ± 0.4	1.6 ± 0.8	**0.034**
**Monocyte infiltration**			
Antrum	1.8 ± 0.4	1.9 ± 0.3	0.500
Corpus	2.2 ± 0.4	1.9 ± 0.5	0.188
**AG**			
Antrum	1.3 ± 0.7	0.6 ± 0.8	0.102
Corpus	0.9 ± 0.8	0.4 ± 0.7	0.273
**IM**			
Antrum	1.0 ± 0.8	0.4 ± 0.7	0.113
Corpus	0.6 ± 0.8	0.1 ± 0.3	0.098
PG I/II ratio	2.0 ± 0.7	5.6 ± 4.3	**0.022**

p-Values were calculated using the chi-square test or Student’s t-test. *Histological grade ≥ 1 atrophic gastritis or intestinal metaplasia and pepsinogen I/II ratio < 2.5. “Dysbiosis” means Acinetobacter predominance after H. pylori eradication. Bold style indicates statistical significance. AG, atrophic gastritis; IM, intestinal metaplasia; PG, pepsinogen

### Follow-Up of Gastric Corpus Mucosa-Associated Microbiota in Subjects With Persistent *H. pylori* Infection

Finally, changes in microbial diversity and bacterial composition were evaluated in patients who failed to eradicate *H. pylori* or who did not receive anti-*Helicobacter* treatment (*n* = 13). There was no significant change in microbial diversity and bacterial composition during follow-up in most patients with persistent infection (Supplementary Figure 9). The changes in bacterial composition at the phylum level in subjects with persistent *H. pylori* infection are presented in Supplementary Figure 10. Interestingly, in one patient who did not receive anti-*Helicobacter* treatment during follow-up, *H. pylori* disappeared spontaneously, with an increase in microbial diversity 10 years later.

## Discussion

In this study, we evaluated gastric microbiota profiles according to *H. pylori* infection and mucosal atrophy/metaplasia in subjects without significant gastrointestinal diseases. The main difference from the previous studies was that gastric corpus microbiota was analyzed instead of antral microbiota. The reason for this approach is based on the results of previous studies (Jo et al., 2016; Sung et al., 2016). That is, non-*H. pylori* bacteria in the antrum have been found to play a minor role in the gastric carcinogenesis (Jo et al., 2016). In contrast, gastric microbiota profiling in the corpus has been suggested as helpful in understanding the role of non-*H. pylori* bacteria in gastric carcinogenesis (Sung et al., 2016). In addition, age-associated changes in gastric microbiota in the corpus, where acid is produced, as well as microbiota changes following *H. pylori* eradication, might have more clinical implications than at the antrum.

First, “healthy” gastric corpus microbiota from *H. pylori*-uninfected subjects without evidence of atrophic gastritis or intestinal metaplasia by histology and pepsinogen I/II ratio ≥ 4.0 (group 1) were distributed in the order of Proteobacteria, Acinetobacter, Firmicutes, and Bacteroidetes (Figure 1A). The distribution was not significantly different from gastric antral microbiota (Bik et al., 2006). Gastric corpus microbiota of *H. pylori*-uninfected individuals showed diversity in bacterial composition. After *H. pylori* infection, however, microbial diversity significantly decreased as *H. pylori* became predominant (Supplementary Figure 4). Our findings are comparable to those of previous studies reporting that following successful *H. pylori* colonization, it dominates the stomach, resulting in a decrease in microbiota diversity (Lee, 2018).

Next, microbiota profiles were compared according to the presence or absence of atrophy/metaplasia. Atrophic gastritis and intestinal metaplasia are considered precancerous lesions in gastric carcinogenesis. Chronic active *H. pylori* infection can lead to these precancerous conditions. When atrophy and intestinal metaplasia occur, however, *H. pylori* abundance decreases in the stomach, and eventually, it is eliminated from the gastric mucosa when severe atrophic gastritis and diffuse intestinal metaplasia occur (Kang et al., 2006). As mucosal atrophy and intestinal metaplasia develop, both gastric pH and microbial diversity increase (Kang et al., 2006; Kusters et al., 2006).

Following the Correa hypothesis, the study subjects were classified into four groups. Group 1 consisted of *H. pylori*-uninfected individuals, and groups 2–4 included *H. pylori*-infected subjects. From groups 2 to 4, the duration of *H. pylori* infection might be increased. Although there was no evidence of active and past *H. pylori* infection and no previous history of *H. pylori* eradication in the subjects of group 4 (*H. pylori*-negative patients with atrophic gastritis or intestinal metaplasia by histology, pepsinogen I/II ratio < 2.5, and *H. pylori* serology negative), *H. pylori* was suggested to have been diminished as mucosal atrophy progressed after a long-standing infection (Kang et al., 2006). Therefore, group 4 may represent a remote past infection of *H. pylori*.

In this study, the gastric microbiota profile of group 4 was compared with that of group 1, and it was found that the relative abundance of *Streptococcus*, *Parvimonas*, and Lactobacillales increased in group 4 (LEfSe analysis, Supplementary Figure 5). These are oral cavity bacteria. Due to hypochlorhydria by mucosal atrophy, many oral cavity bacteria can colonize the stomach, and thus, the gastric microbiota becomes similar to oral cavity microbiota (Sheh and Fox, 2013). An increase in the abundance of *Streptococcus* species has been previously reported to be associated with the development of gastric cancer (Bik et al., 2006). Furthermore, the relative abundance of both non-*H. pylori* urease-producing bacteria as well as non-*H. pylori* nitrosating bacteria was significantly increased in group 4 (Supplementary Figure 3). It is well known that many non-*H. pylori* urease-producing bacteria and nitrosating or nitrate-reducing bacteria other than *H. pylori* exist in the stomach (Mowat et al., 2000; Sanduleanu et al., 2001). Nitrosating bacteria and urease-producing bacteria play an important role in gastric carcinogenesis (Peng et al., 2017).

In the present study, *H. pylori*-uninfected individuals (group 1) were followed up to 10 years. Based on our current knowledge, this is the first study evaluating the time course of changes in gastric microbiota profiles in *H. pylori*-uninfected individuals. We found that the relative abundance of Proteobacteria (*Enhydrobacter*, Comamonadaceae, *Sphingobium*) increased; that of Firmicutes (*Streptococcus*, *Veillonella*), Fusobacteria (*Fusobacterium*), Nocardioidaceae, *Rothia*, and *Prevotella* decreased; and microbial diversity decreased with age (Figure 3, Supplementary Figure 6, and Supplementary Table 2). These microbiota changes in the stomach might be a phenomenon of aging. Our findings are important especially when the gastric microbiota profiles were compared between gastric cancer cases and controls; the effect of aging should be considered before the interpretation of the microbiome data because age is not only a risk factor for gastric cancer but also has an effect on gastric microbiota (Ferreira et al., 2018).

In this study, we investigated long-term changes in the gastric microbiota following *H. pylori* eradication (*n* = 21). In 10 out of 21 patients (47.6%), the gastric microbiota appeared to be restored to that of *H. pylori*-negative individuals (without *Acinetobacter* predominance group, Supplementary Figure 5). However, in 11 patients (52.4%), severe dysbiosis with an increase in *Actinobacteria* abundance occurred, and the microbial diversity decrease by *H. pylori* infection was not restored (*Acinetobacter* predominance group). Our findings suggest that *H. pylori* eradication does not always normalize the gastric microbiota.

Antibiotics used for *H. pylori* eradication can alter the gut microbiota, at least temporarily (Iizumi et al., 2017). A recent study has reported significant changes in the gut microbiota structure and an increase in antibiotic resistance by quadruple *H. pylori* eradication therapy (Olekhnovich et al., 2019). In most of the studies, however, gut microbiota changes caused by antibiotic treatment were reversible, and the restoration of gut microbiota a few weeks after has been reported (He et al., 2019; Liou et al., 2019). On the other hand, there are a few studies regarding the changes in gastric mucosa-associated microbiota after *H. pylori* eradication (Li et al., 2017; Guo and Zhang, 2020; Gantuya and El Serag, 2020). A pilot study has reported that *H. pylori*-induced gastric microbiota alternations can be recovered by anti-*Helicobacter* eradication treatment (Li et al., 2017). A recent Chinese study has also shown that successful *H. pylori* eradication potentially restores gastric microbiota to a status similar to that found in uninfected individuals and has beneficial effects on gut microbiota (Guo and Zhang, 2020). However, a recent prospective trial has reported that increased abundance of *Acinetobacter lwoffii*, *Streptococcus anginosus*, and *Ralstonia* was associated with persistent inflammation, and that of *Granulicatella*, *Actinomyces*, *Rothia*, *Peptostreptococcus*, *Streptococcus*, *Abiotrophia*, and *Parvimonas* was associated with atrophy/metaplasia in patients 1 year following successful *H. pylori* eradication; most of the significant taxa were oral cavity microbes and formed a distinct cluster in the microbial ecology in the absence of *H. pylori* (Sung et al., 2020). Therefore, additional studies are necessary to clarify this issue.

In the present study, we obtained data that could explain the difference between the two *H. pylori* eradication groups (Table 2). The proportion of individuals with atrophic gastritis/intestinal metaplasia at baseline was significantly different between the two groups (*p* = 0.011 by χ^2^ test). In addition, the increased abundance of *Acinetobacter* at baseline was significantly associated with dysbiosis after anti-*Helicobacter* treatment (*p* = 0.005, Table 2). One possible explanation is that persistent hypochlorhydria in subjects with severe atrophic gastritis even after *H. pylori* eradication may lead to colonization of the stomach with various bacteria, which can suppress the colonization of *Acinetobacter* species. In contrast, *H. pylori* eradication can restore normal gastric acid secretion in individuals without atrophic gastritis. Low gastric pH promotes an environment unfavorable for the survival of most bacteria. In contrast, some *Acinetobacter* species resistant to anti-*Helicobacter* treatment can survive and become predominant in this acidic environment. Some *Acinetobacter* species, such as *A. lwoffii* and *A. anitratu*, can produce urease under appropriate conditions (Enjalbert et al., 1959). A recent study has reported that the abundance of *Acinetobacter*, especially *A. lwoffii*, *A. rhizosphaerae*, and *A. guillouiae*, increased after *H. pylori* eradication (Sung et al., 2020). An increase in *Acinetobacter* species abundance was associated with persistent gastric inflammation and negatively correlated with the histological grade of atrophy and intestinal metaplasia after successful eradication, which is consistent with our findings (Sung et al., 2020).

Our findings suggest that gastric dysbiosis after *H. pylori* eradication is not temporary but persists for years. In addition, the colonization of *Acinetobacter* species in a subset of patients carries the potential risk for antibiotic-resistant bacterial infection (Munoz-Price and Weinstein, 2008; Doughari et al., 2011). However, the impact of dysbiosis on health and disease could be evaluated in this study. Therefore, further research is necessary to address this issue.

In this study, there were no changes in microbial diversity and bacterial composition in persistently infected individuals (Supplementary Figure 9). However, in one subject (C93, 67-year-old woman) with atrophic gastritis and intestinal metaplasia at baseline, *H. pylori* infection spontaneously regressed with an increased microbial diversity after 11 years of follow-up (Supplementary Figure 10). At that time, she was diagnosed with early gastric cancer, which was successfully treated with endoscopic resection. The case shows dynamic gastric microbiota changes and the consequences of long-standing *H. pylori* infection.

The present study has several limitations that should be considered. First, the sample size of the study was not sufficient to draw conclusions for some issues, such as changes in gut microbiota due to the *H. pylori* eradication regimen. In addition, the sample size of subgroups was inhomogeneous. Especially, the number of patients in group 4 was so small (*n* = 3), and all of them were women. Therefore, the positive findings for group 4 should be validated through further studies. Second, only gastric corpus mucosal samples, not gastric antral mucosal samples, were analyzed. However, we have previously reported that the gastric microbiota profile at the antrum did not appear to have a major role in gastric carcinogenesis (Jo et al., 2016). Third, follow-up durations and intervals were inhomogeneous. Korea has the highest incidence rates for gastric cancer in the world; gastroscopy is covered by national insurance biennially for individuals over 40 years old. In addition, endoscopy is recommended for individuals with dyspeptic symptoms or a family history of stomach cancer. In this study, we recommended that gastroscopy be performed at intervals of 2 years (in case of no atrophy, intestinal metaplasia, and *H. pylori*-negative) or 1 year (in case of severe atrophy/IM or family history of gastric cancer) in the outpatient clinic. However, it was an observational study; thus, follow-up endoscopy was not mandatory. Fourth, the study subjects were from 26 to 70 years old at the time of enrollment, so there are no data on individuals under 20 years when most individuals in developing countries are infected by *H. pylori.* Therefore, caution should be taken in the interpretation of the changes of gastric microbiota with aging. Despite these limitations, our findings are derived from a long-term follow-up of more than 10 years and provided valuable information on age-dependent changes in *H. pylori*-negative gastric mucosa.

## Conclusion

In conclusion, in *H. pylori*-uninfected gastric corpus mucosa, the relative abundance of Proteobacteria increases, relative abundance of Firmicutes and Fusobacteria decreases, and microbial diversity decreases with aging. After successful *H. pylori* eradication, gastric microbiota can be restored in some individuals, but it may not always be restored by anti-*Helicobacter* treatment.

## Data Availability Statement

The raw unprocessed gene datasets of 16S rRNA, which were generated during the current study, are available with the NCBI Sequence Read Archive (SUB7790380, SRA: PRJNA647534), https://www.ncbi.nlm.nih.gov/sra/?term=PRJNA647534 (accession Numbers: SRR12278303-SRR12278401).

## Ethics Statement

The studies involving human participants were reviewed and approved by the Institutional Review Board of Seoul National University Bundang Hospital (B-1903/529-302). The patients/participants provided their written informed consent to participate in this study.

## Author Contributions

CS performed clinical and microbiome data analysis, and drafted and revised the article. NK conceptualized the study, enrolled the study participants, and revised the manuscript. JP performed the experiments including DNA preparation from the gastric mucosal samples. DL received the research grant and revised the manuscript. All authors approved the final version of the manuscript before submission.

## Conflict of Interest

The authors declare that the research was conducted in the absence of any commercial or financial relationships that could be construed as a potential conflict of interest.

## Supplementary Material

Supplementary Figure 1 Study flow chart.

Supplementary Figure 2 Bacterial composition of the study subjects (*N* = 43) at phylum level.

Supplementary Figure 3 Relative abundance of nitrosating bacteria other than *H. pylori* **(A)** and urease-producing bacteria other than *H. pylori* **(B)** at gastric corpus mucosae. In Group 4, relative abundance of nitrosating bacteria other than *H. pylori* was increased (ANOVA *p* < 0.05, **A**). In Group 4, relative abundance of urease-producing bacteria other than *H. pylori* was significantly increased compared to other groups (ANOVA *p* < 0.05, **B**). Group 1: *H. pylori*-uninfected subjects without evidence of atrophic gastritis and intestinal metaplasia by histology, pepsinogen I/II ratio ≥ 4.0, and no history of *H. pylori* eradication (*n* = 8), Group 2: *H. pylori*-infected patients without mucosal atrophy and metaplasia by histology with pepsinogen I/II ratio > 4.0 (*n* = 15), Group 3: *H. pylori*-infected patients with atrophic gastritis and/or intestinal metaplasia by histology and pepsinogen I/II ratio < 2.5 (*n* = 17), Group 4: the patients with remote past *H. pylori* infection without history of *H. pylori* eradication (*n* = 3). *Hp, Helicobacter pylori*.

Supplementary Figure 4 Gastric corpus mucosa-associated microbiome changes by *H. pylori* infection. LEfSe analyses showed an increase in Proteobacteria abundance and a decrease in Actinobacteria, Sphingomonadales, *Prevotella*, and *Veillonella* abundance **(A)**. Alpha-diversity indices were significantly decreased in *H. pylori-*infected patients **(B)**.

Supplementary Figure 5 Summary of the linear discriminant analysis (LEfSe) comparing Group 1 with Group 4. The figure shows significantly different taxa (*p* < 0.05 and Log10 (LDA score) > 3.0). Among *H. pylori*-negative subjects (*n* = 11), the relative abundance of Firmicutes, including *Streptococcus*, *Parvimonas*, and Lactobacillales, was increased in Group 4 compared with Group 1. In contrast, the relative abundance of alphaproteobacteria (*Methylobacterium* and *Paracoccus*), Chloroflexi (Thermomicrobia), Corynebacteriales, Microbacteriaceae, Propionibacteriaceae (*Cutibacterium*), and Burkholderiales (*Massilia, Diaphorobacter*, and *Comamonadaceae*) was decreased in Group 4. Group 1: *H. pylori*-uninfected subjects without evidence of atrophic gastritis and intestinal metaplasia by histology, pepsinogen I/II ratio ≥ 4.0, and no history of *H. pylori* eradication (*n* = 8), Group 4: the patients with atrophy/metaplasia, no evidence of active *H. pylori* infection, negative for anti-*H. pylori* Ig G antibody test, and no previous history of *H. pylori* eradication (*n* = 3).

Supplementary Figure 6 Changes of gastric microbiota during the follow-up in the *H. pylori*-negative/non-atrophy subjects (*n* = 8). At family level, an increase of Moraxellaceae and Comamonadaceae abundance and a decrease of Firmicutes, Streptococcaceae, Prevotellaceae, Veillonellaceae, Nocardioidaceae, Fusobacteriaceae, and Actinomycetaceae abundance were observed during the follow-up (FDR *q*-value < 0.05, **A**). At genus level, the relative abundance of *Enhydrobacter*, *Sphingobium* and *Chryseobacterium* was increased and that of *Streptococcus*, *Prevotella*, *Veillonella*, *Rothia* and *Fusobacterium* was decreased (FDR *q*-value < 0.05, **B**). FDR, false discovery rate.

Supplementary Figure 7 Changes in gastric microbiota at the corpus in *H. pylori*-uninfected individuals without atrophic gastritis/intestinal metaplasia (Group 1, *n* = 8).

Supplementary Figure 8 Changes in gastric microbiota after *H. pylori* eradication. In 10 patients both microbiota composition and microbial diversity appeared to be restored **(A)**, but in 11 patients they were not restored **(B)**.

Supplementary Figure 9 Changes in gastric microbiota in subjects with persistent *H. pylori* infection. *A -diversity indices* (Shannon index, **A**; Phylogenetic diversity, **B**) and bacterial composition **(C)** were not changed significantly.

Supplementary Figure 10 Gastric microbiota in patients with persistent *H. pylori* infection.
