# Supplementary figures and images for: Changes in Gastric Corpus Microbiota With Age and After Helicobacter pylori Eradication: A Long-Term Follow-Up Study
Source: Front Microbiol. 2021 Feb 9;11:621879. doi: 10.3389/fmicb.2020.621879 (PMC7900007; doi:10.3389/fmicb.2020.621879)

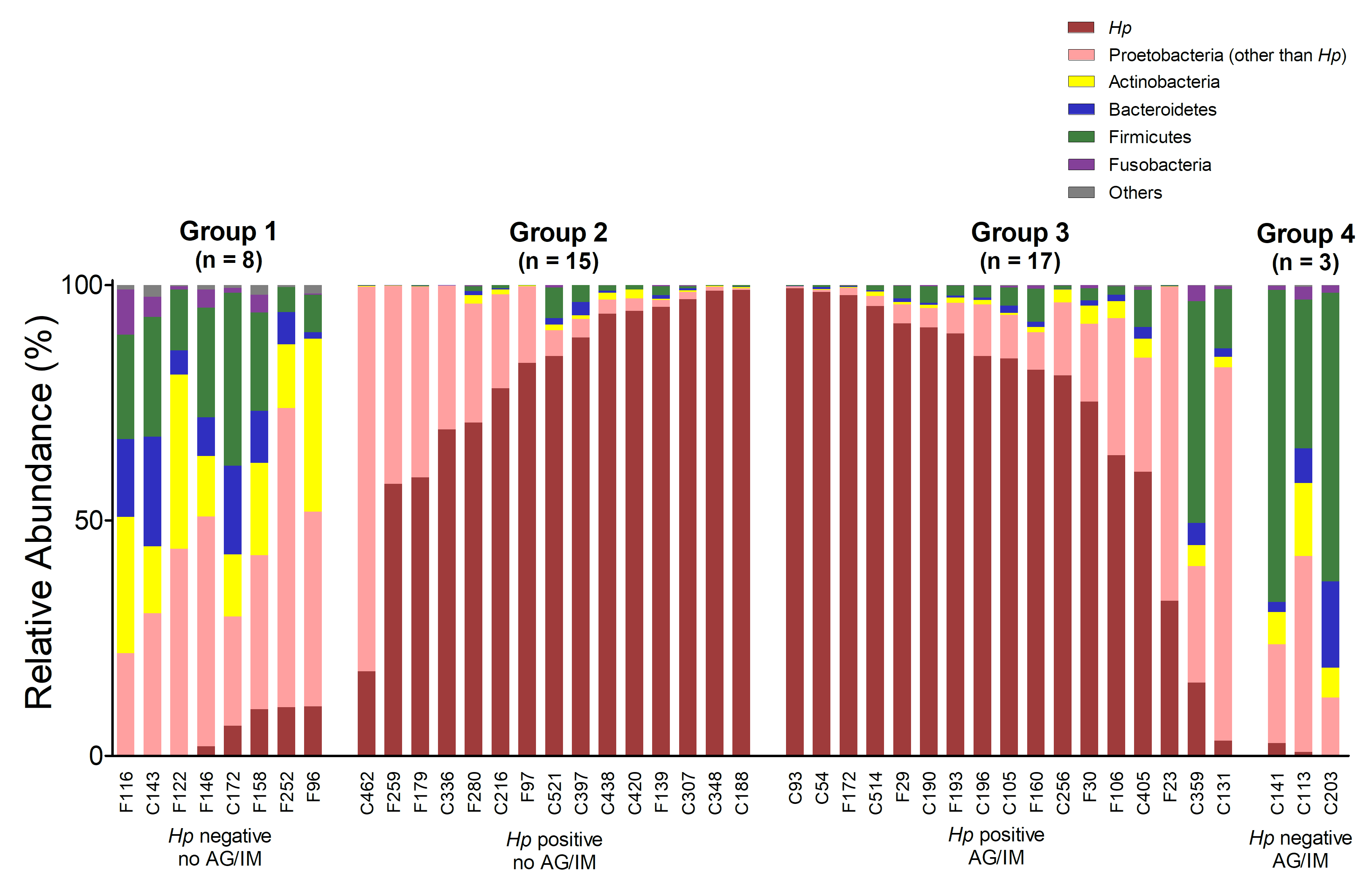

Supplement: Supplementary Figure 2 — Bacterial composition of the study subjects (N = 43) at phylum level. [file Image_2.TIF]

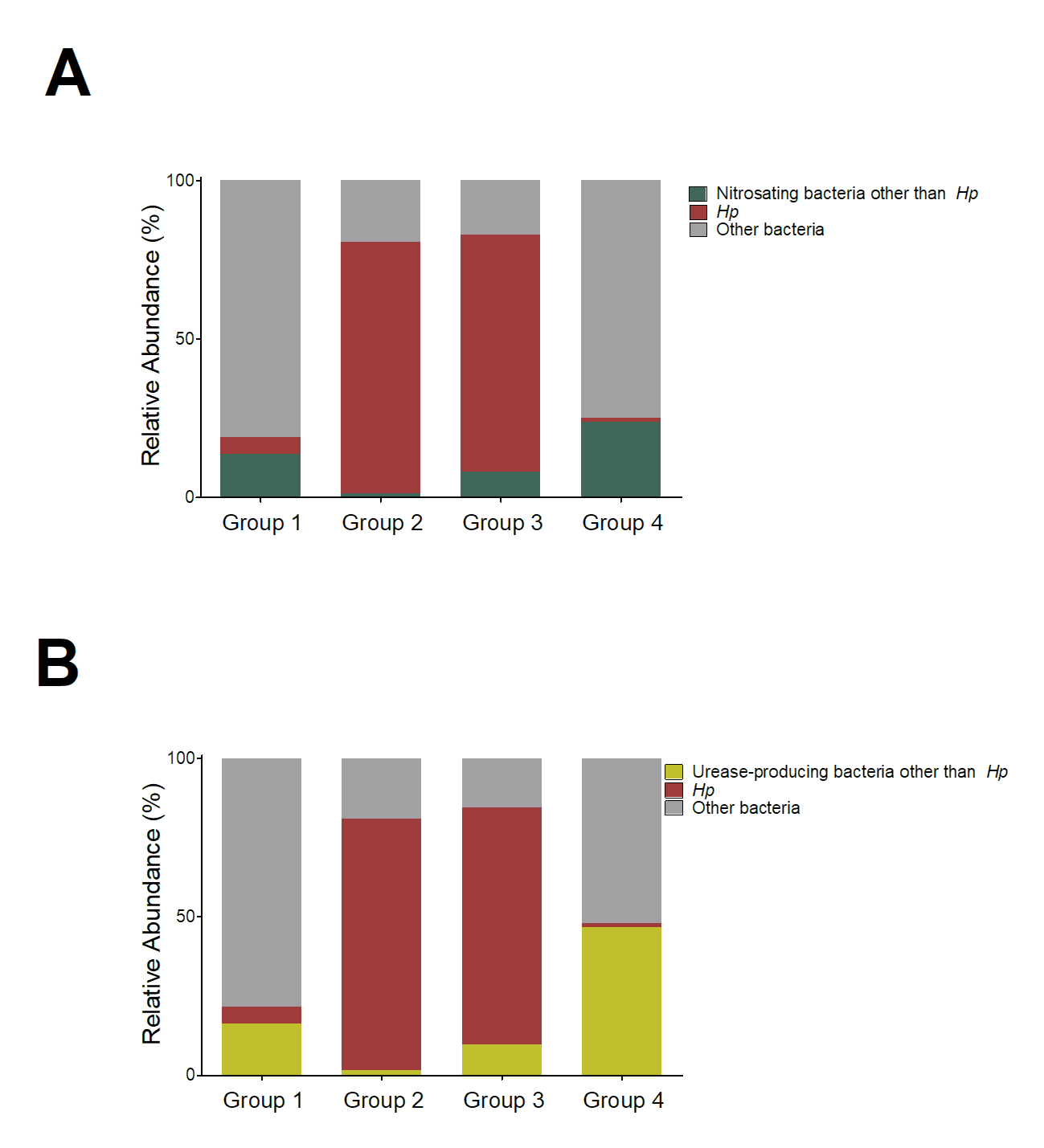

Supplement: Supplementary Figure 3 — Relative abundance of nitrosating bacteria other than H. pylori (A) and urease-producing bacteria other than H. pylori (B) at gastric corpus mucosae. In Group 4, relative abundance of nitrosating bacteria other than H. pylori was increased (ANOVA p < 0.05, A). In Group 4, relative abundance of urease-producing bacteria other than H. pylori was significantly increased compared to other groups (ANOVA p < 0.05, B). Group 1: H. pylori-uninfected subjects without evidence of atrophic gastritis and intestinal metaplasia by histology, pepsinogen I/II ratio ≥ 4.0, and no history of H. pylori eradication (n = 8), Group 2: H. pylori-infected patients without mucosal atrophy and metaplasia by histology with pepsinogen I/II ratio > 4.0 (n = 15), Group 3: H. pylori-infected patients with atrophic gastritis and/or intestinal metaplasia by histology and pepsinogen I/II ratio < 2.5 (n = 17), Group 4: the patients with remote past H. pylori infection without history of H. pylori eradication (n = 3). Hp, Helicobacter pylori. [file Image_3.TIF]

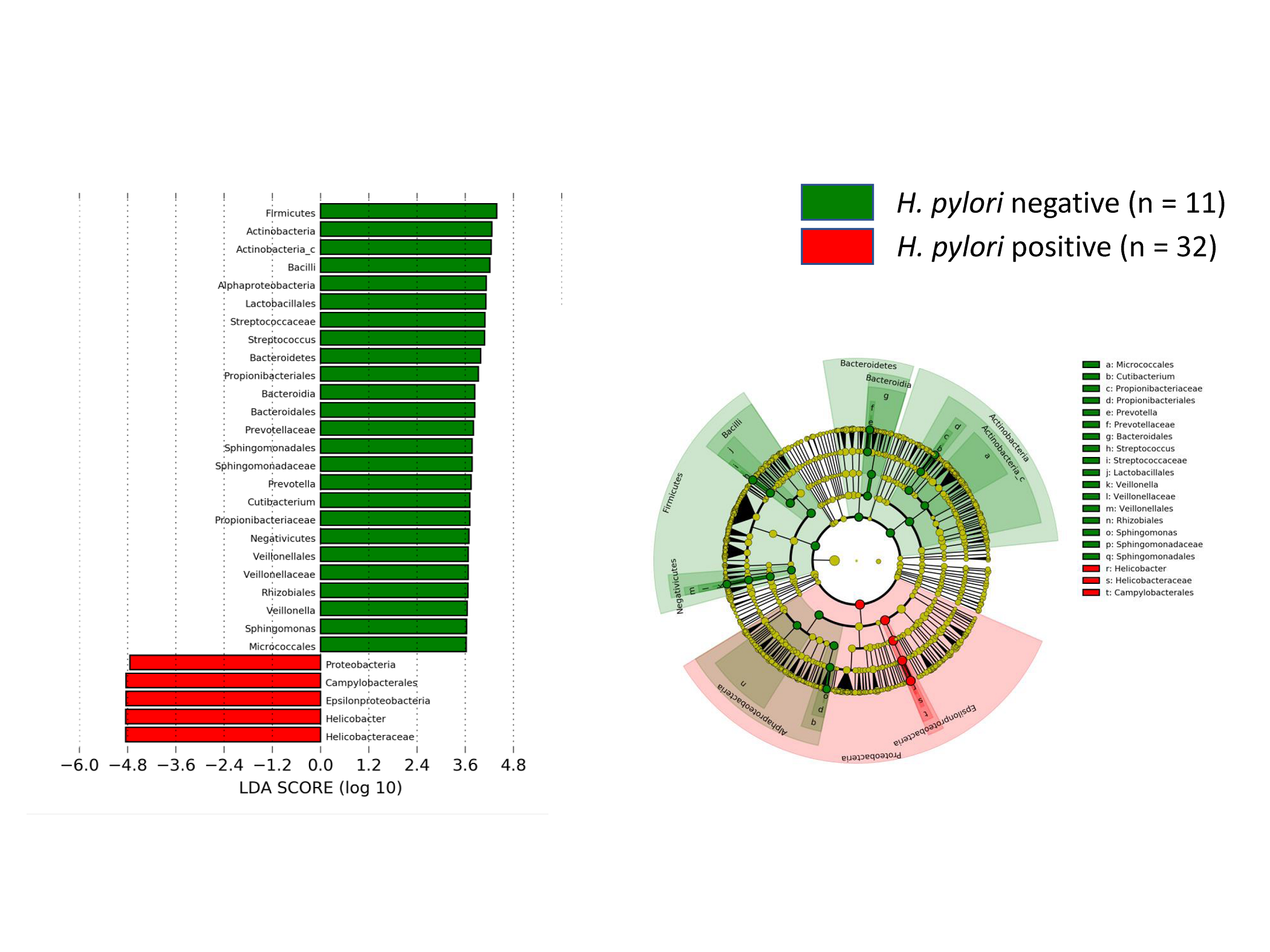

Supplement: Supplementary Figure 4 — Gastric corpus mucosa-associated microbiome changes by H. pylori infection. LEfSe analyses showed an increase in Proteobacteria abundance and a decrease in Actinobacteria, Sphingomonadales, Prevotella, and Veillonella abundance (A). Alpha-diversity indices were significantly decreased in H. pylori-infected patients (B). [file Image_4.TIF]

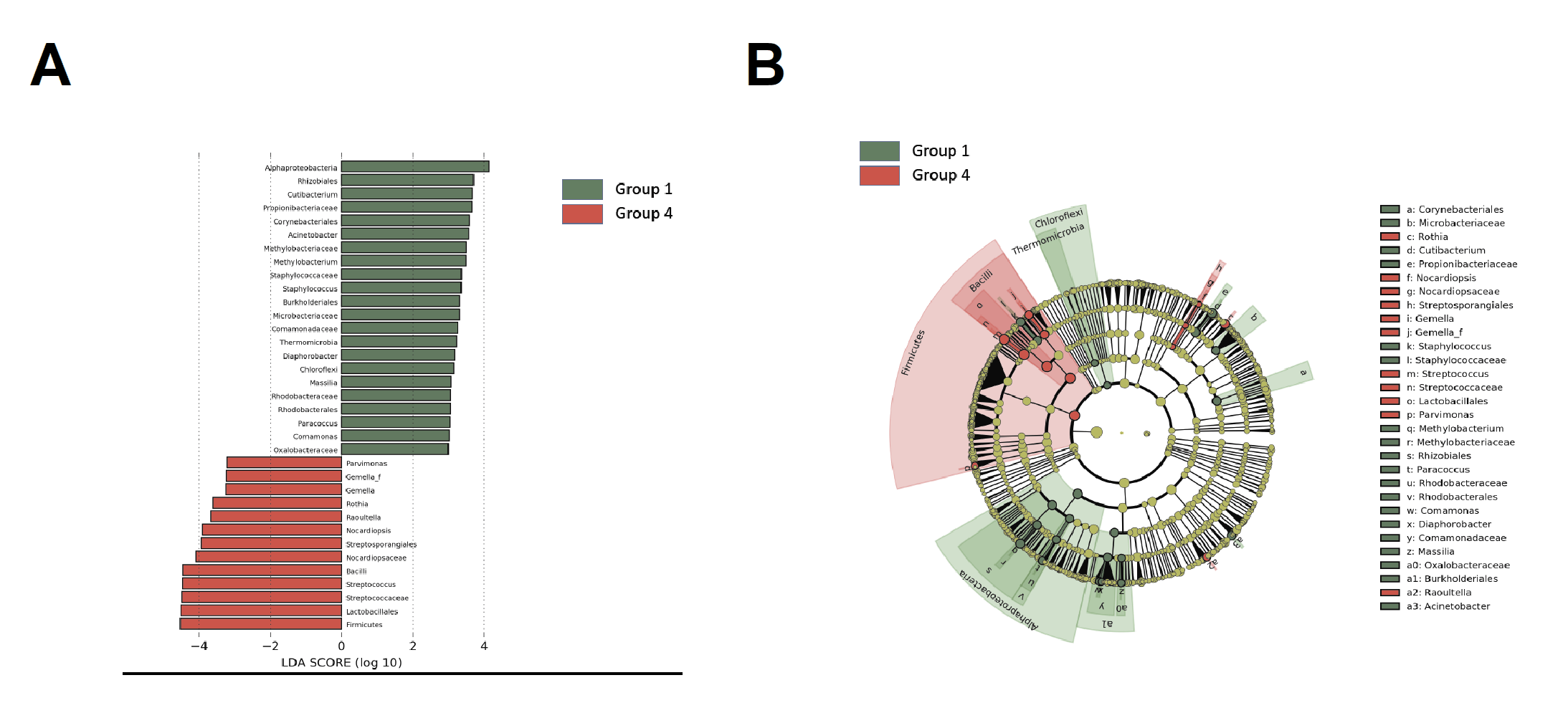

Supplement: Supplementary Figure 5 — Summary of the linear discriminant analysis (LEfSe) comparing Group 1 with Group 4. The figure shows significantly different taxa (p < 0.05 and Log10 (LDA score) > 3.0). Among H. pylori-negative subjects (n = 11), the relative abundance of Firmicutes, including Streptococcus, Parvimonas, and Lactobacillales, was increased in Group 4 compared with Group 1. In contrast, the relative abundance of alphaproteobacteria (Methylobacterium and Paracoccus), Chloroflexi (Thermomicrobia), Corynebacteriales, Microbacteriaceae, Propionibacteriaceae (Cutibacterium), and Burkholderiales (Massilia, Diaphorobacter, and Comamonadaceae) was decreased in Group 4. Group 1: H. pylori-uninfected subjects without evidence of atrophic gastritis and intestinal metaplasia by histology, pepsinogen I/II ratio ≥ 4.0, and no history of H. pylori eradication (n = 8), Group 4: the patients with atrophy/metaplasia, no evidence of active H. pylori infection, negative for anti-H. pylori Ig G antibody test, and no previous history of H. pylori eradication (n = 3). [file Image_5.TIF]

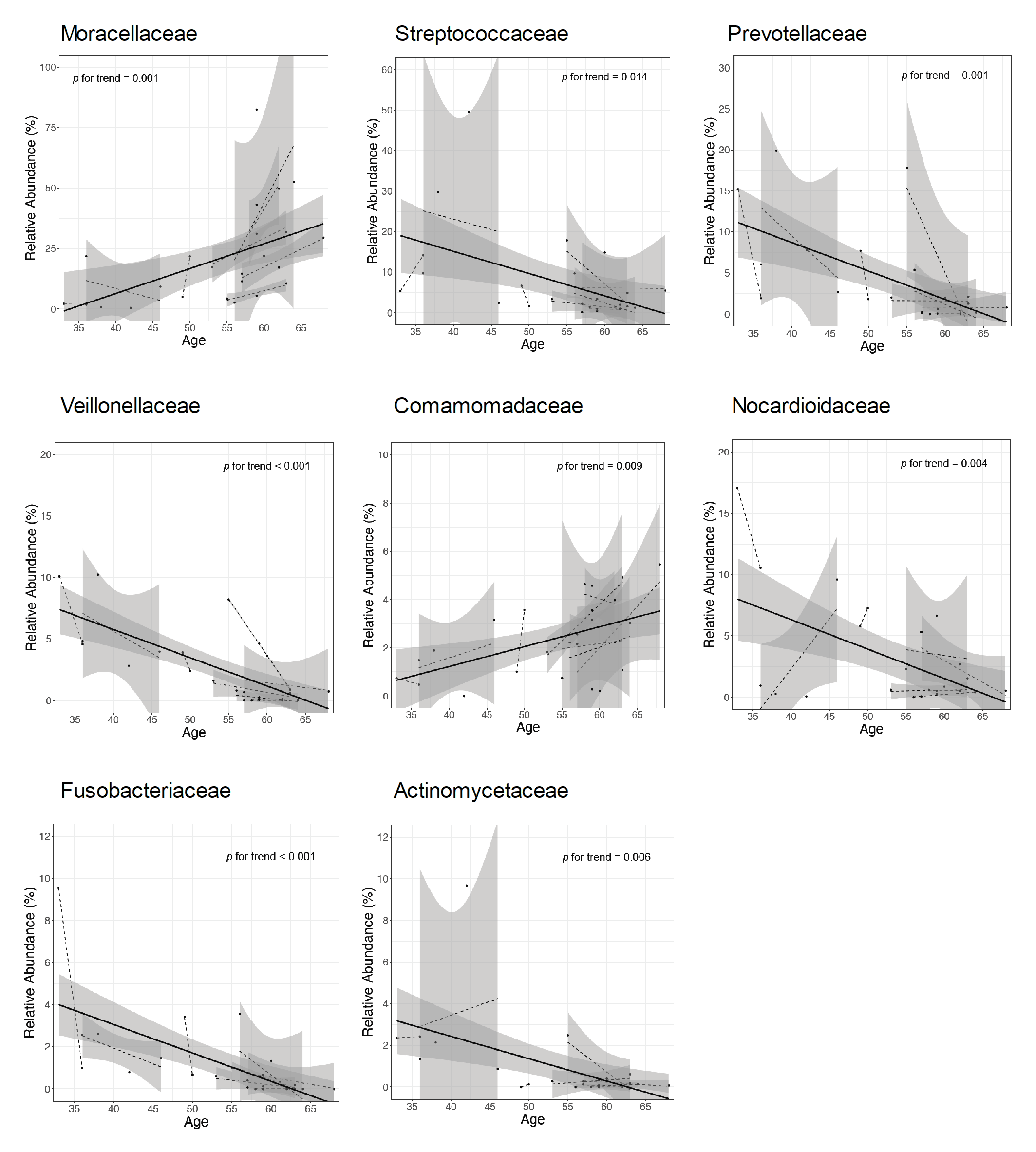

Supplement: Supplementary Figure 6 — Changes of gastric microbiota during the follow-up in the H. pylori-negative/non-atrophy subjects (n = 8). At family level, an increase of Moraxellaceae and Comamonadaceae abundance and a decrease of Firmicutes, Streptococcaceae, Prevotellaceae, Veillonellaceae, Nocardioidaceae, Fusobacteriaceae, and Actinomycetaceae abundance were observed during the follow-up (FDR q-value < 0.05, A). At genus level, the relative abundance of Enhydrobacter, Sphingobium and Chryseobacterium was increased and that of Streptococcus, Prevotella, Veillonella, Rothia and Fusobacterium was decreased (FDR q-value < 0.05, B). FDR, false discovery rate. [file Image_6.TIF]

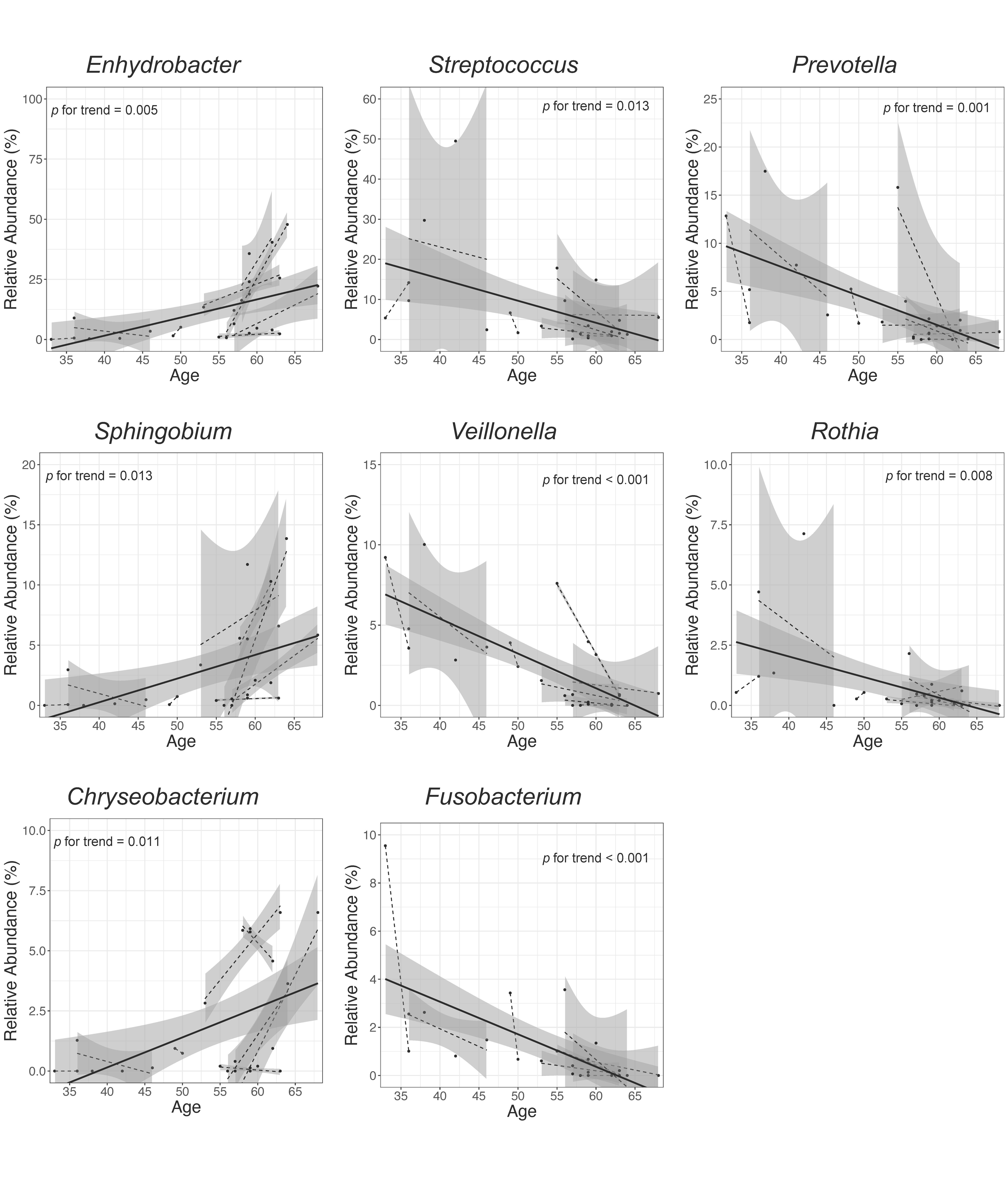

Supplement: Supplementary Figure 7 — Changes in gastric microbiota at the corpus in H. pylori-uninfected individuals without atrophic gastritis/intestinal metaplasia (Group 1, n = 8). [file Image_7.TIFF]

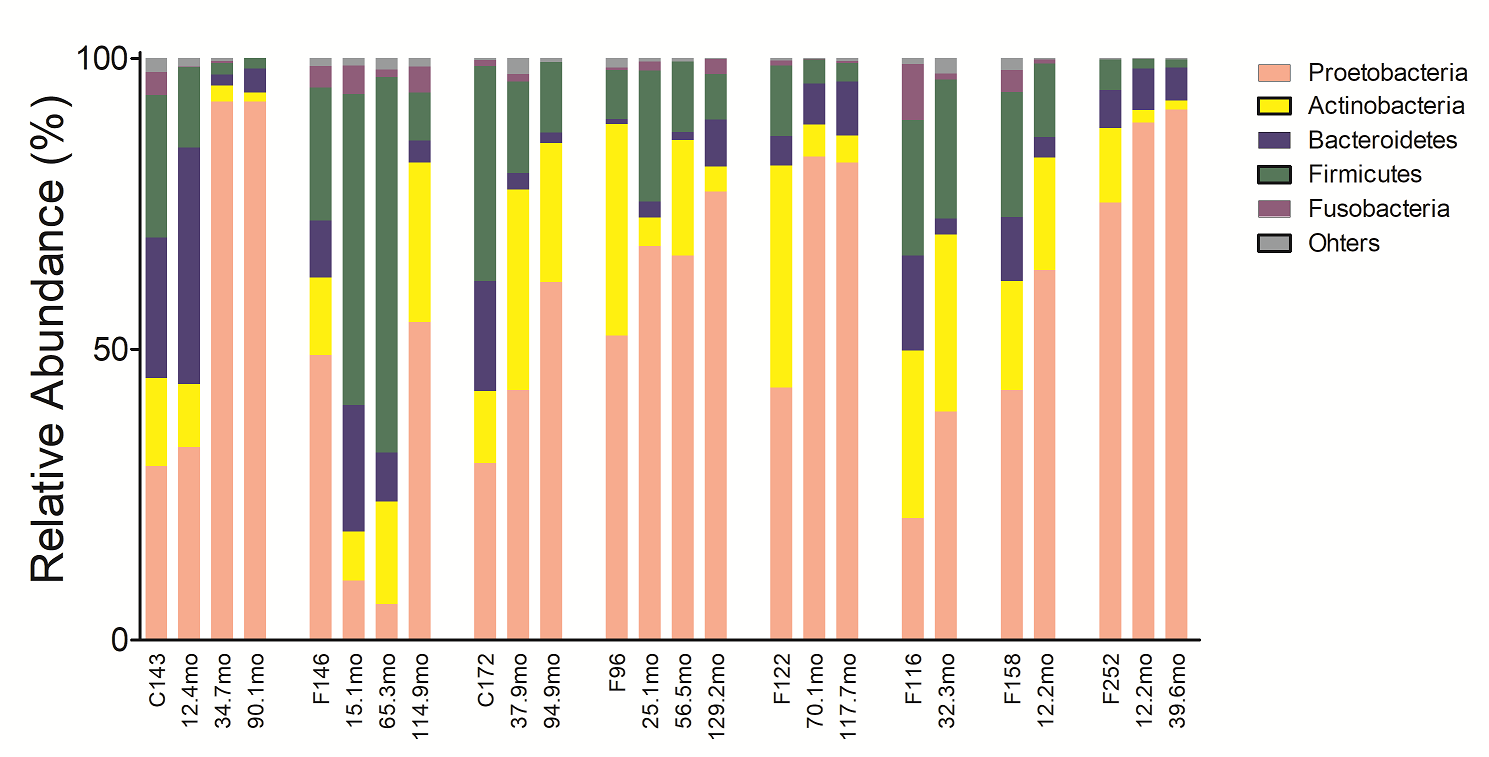

Supplement: Supplementary Figure 8 — Changes in gastric microbiota after H. pylori eradication. In 10 patients both microbiota composition and microbial diversity appeared to be restored (A), but in 11 patients they were not restored (B). [file Image_8.TIF]

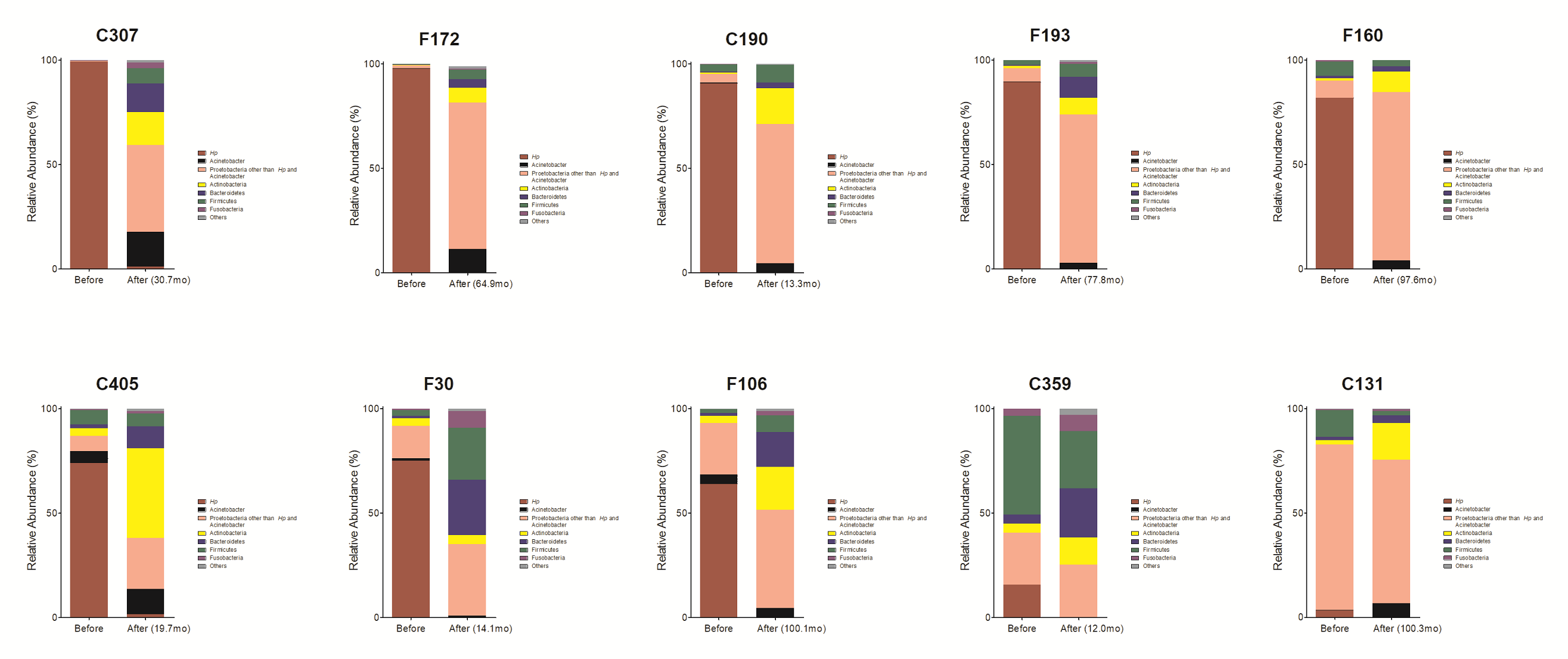

Supplement: Supplementary Figure 9 — Changes in gastric microbiota in subjects with persistent H. pylori infection. A -diversity indices (Shannon index, A; Phylogenetic diversity, B) and bacterial composition (C) were not changed significantly. [file Image_9.TIF]

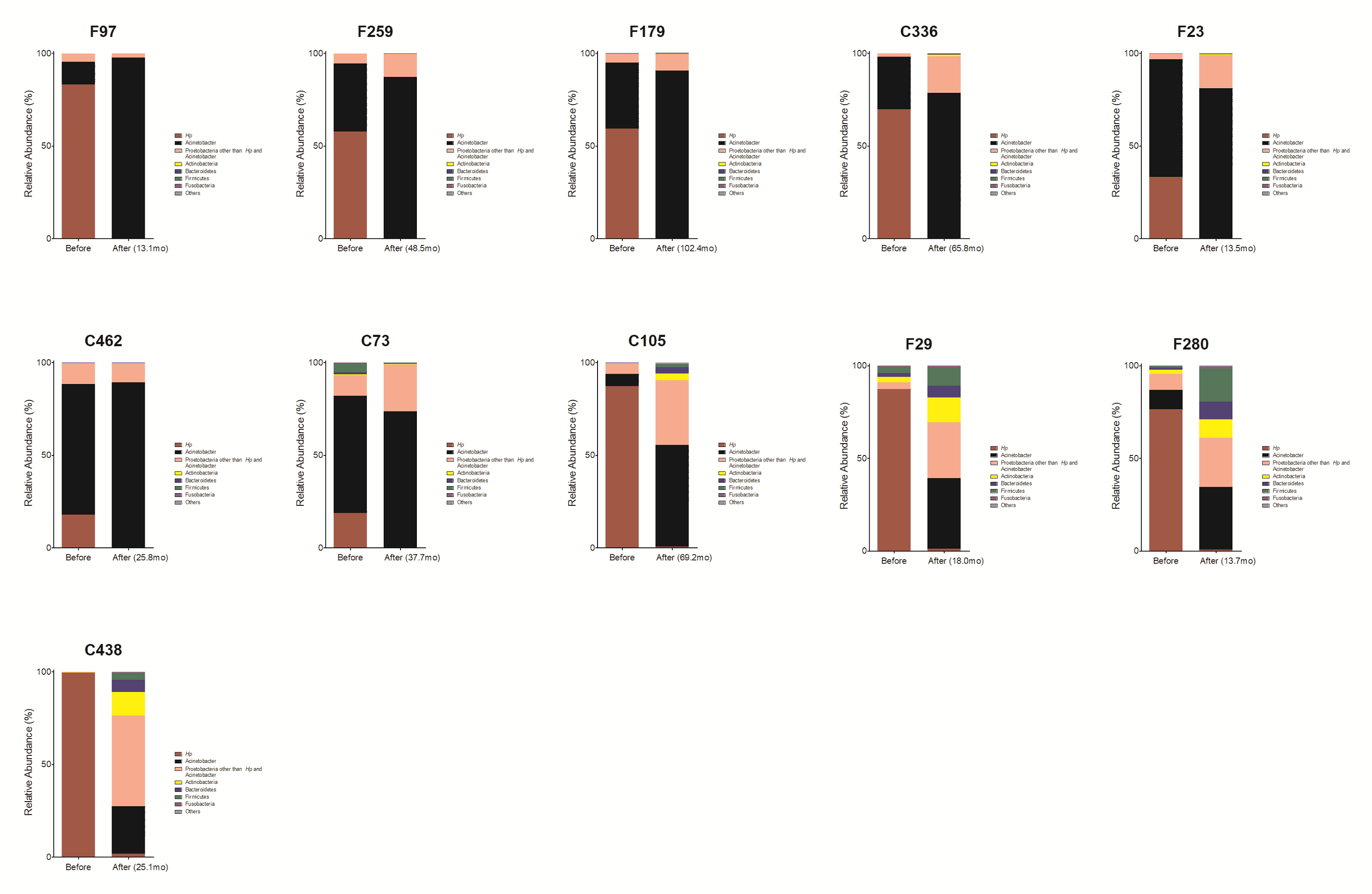

Supplement: Supplementary Figure 10 — Gastric microbiota in patients with persistent H. pylori infection. [file Image_10.TIF]

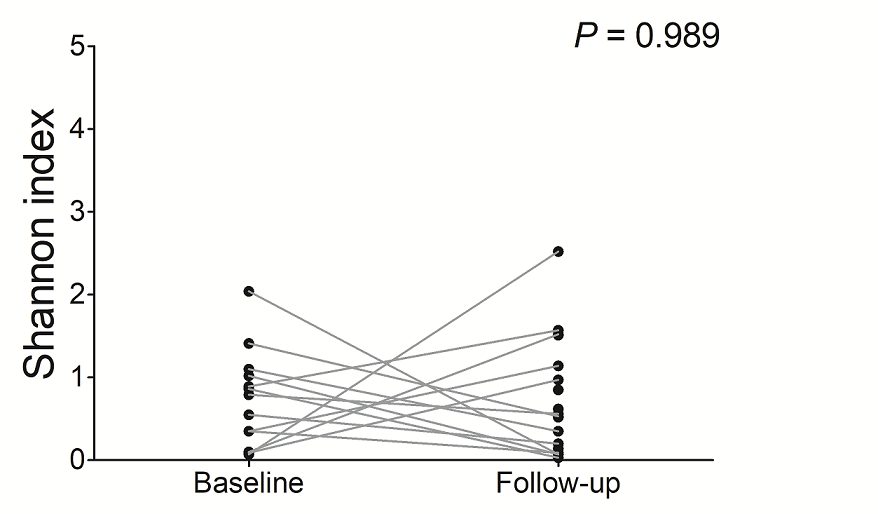

Supplement: Supplementary file 11 [file Image_11.TIF]

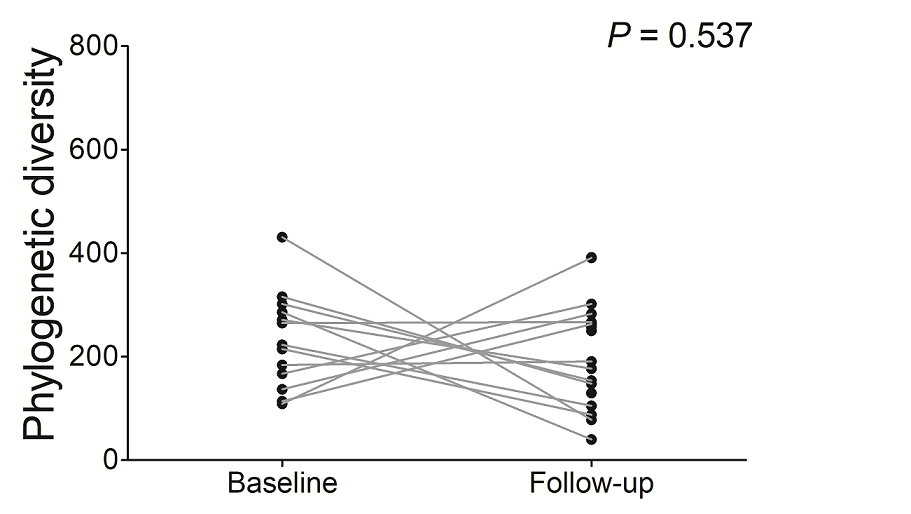

Supplement: Supplementary file 12 [file Image_12.TIF]

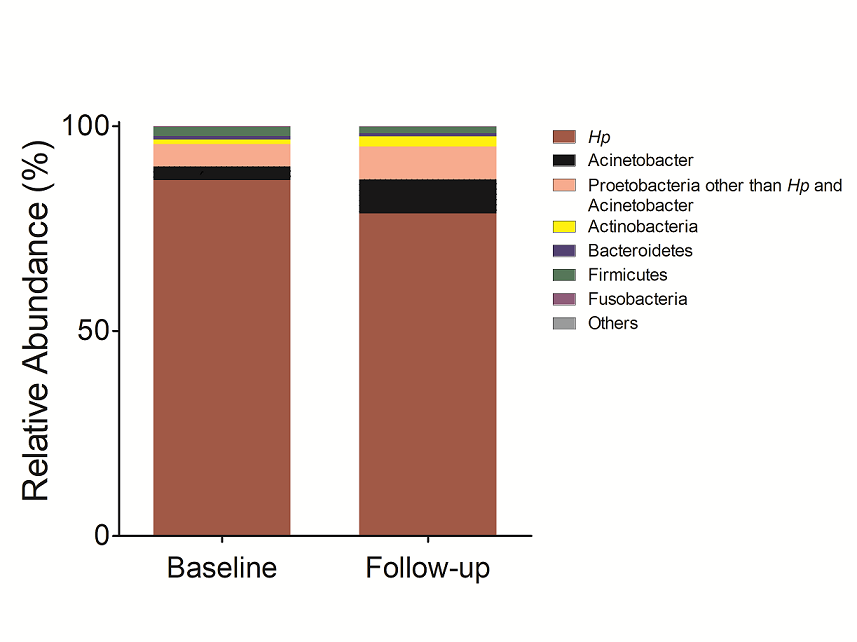

Supplement: Supplementary file 13 [file Image_13.TIF]

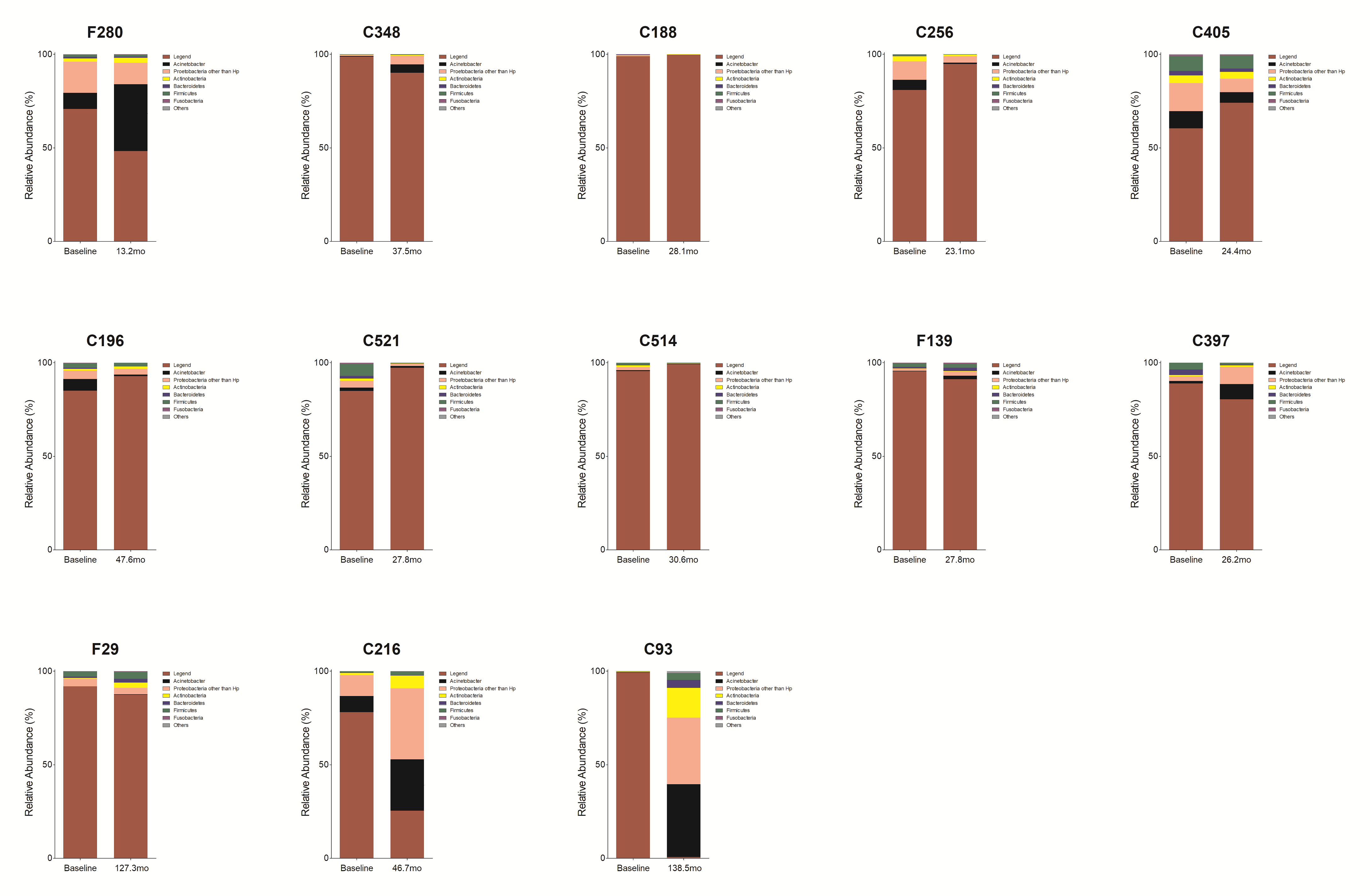

Supplement: Supplementary file 14 [file Image_14.TIF]
