# Supplementary material for: Changes in Gastric Corpus Microbiota With Age and After Helicobacter pylori Eradication: A Long-Term Follow-Up Study
Source: Front Microbiol. 2021 Feb 9;11:621879. doi: 10.3389/fmicb.2020.621879 (PMC7900007; doi:10.3389/fmicb.2020.621879)
Supplement: Supplementary file 15 [file Table_1.PDF]

Supplementary Table S1. List of nitrosating bacteria and urease-producing bacteria

Species of nitrosating bacteria (nitrate-reducing bacteria)	Species of urease-producing bacteria
Helicobacter pylori	*Helicobacter pylori*
Bacteroides fragilis	*Helicobacter bizzozeronii*
Bacteroides plebeius	*Helicobacter felis*
Campylobacter uc	*Actinobacillus hominis*
Citrobacter freundii	*Actinobacillus suis*
Clostridium bartlettii	*Acitnobacillus ureae*
Clostridium bifermentans	*Actinomyces naeslundii*
Clostridium g4_uc	*Actinomyces suis*
Clostridium lituseburense	*Actinomyces viscosus*
Clostridium perfringens	*Bordetella bronchiseptica*
Clostridium sordellii	*Bordetella parapertusis*
Corynebacterium accolens	*Brucella abortus*
Corynebacterium afermentans	*Brucella melitensis*
Corynebacterium amycolatum	*Brucella suis*
Corynebacterium argentoratense	*Campylobacter hyointestinalis*
Corynebacterium durum	*Campylobacter sputorum*
Corynebacterium glucuronolyticum	*Citrobacter amalonaticus*
Corynebacterium kroppenstedtii	*Citrobacter freundii*
Corynebacterium macginleyi	*Citrobacter rodentium*
Corynebacterium matruchotii	*Clostridium perfringens*
Corynebacterium mucifaciens	*Clostridium sordellii*
Corynebacterium mycetoides	*Corynebacterium pseudodiphthericum*
Corynebacterium pilbarense	*Corynebacterium ulcerans*
Corynebacterium pseudogenitalium	*Corynebacterium urealyticum*
Corynebacterium singulare	*Cryptococcus albidus*
Corynebacterium suicordis	*Cryptococcus laurentii*
Corynebacterium thomssenii	*Cryptococcus noeformans*
Corynebacterium tuberculostearicum_group	*Enterobacter asburiae*
Corynebacterium uc	*Enterobacter cloacae*
Corynebacterium ureicelerivorans	*Enterobacter gergoviae*
Corynebacterium variabile	*Haemophilus influenzae*
Corynebacterium vitaeruminis	*Haemophilus parainfluenzae*
Corynebacterium xerosis	*Haemophilus parahaemolyticus*
Escherichia coli	*Klebsiella oxytoca*
Haemophilus influenzae	*Klebsiella planticola*
Haemophilus parainfluenzae	*Klebsiella pneumoniae*
Klebsiella pneumoniae	*Micrococcus tetragenus*
Lactobacillus fermentum	*Morganella morganii*
Lactobacillus gasseri	*Mycobacterium africanum*
Neisseria bacilliformis	*Mycobacterium genavense*
Neisseria cinerea	*Mycobacterium heidelbergense*
Neisseria elongata	*Pasteurella aerogenes*
Neisseria flava	*Pasteurella dagmatis*
Neisseria flavescens	*Pasteurella pneumotropica*
Neisseria macacae	*Proteus mirabilis*
Neisseria mucosa	*Proteus myxofaciens*
Neisseria oralis	*Proteus vulgaris*
Neisseria perflava	*Providencia rettgeri*
Neisseria sicca	*Providencia stuartii*
Neisseria sicca group	*Pseudomonas aeruginosa*
Neisseria subflava	*Pseudomonas monteilii*
Neisseria uc	*Pseudomonas mendocina*
Pseudomonas aeruginosa	*Staphylococcus epidemidis*
Pseudomonas stutzeri	*Staphylococcus hominis*
Staphylococcus aureus	*Staphylococcus saprophyticus*
Staphylococcus epidermidis	*Streptococcus salivarius*
Staphylococcus hominis	*Streptococcus thermophlilis*
Staphylococcus aprophyticus	*Ureaplasma urealyticum*
Stenotrophomonas maltophilia	*Yersinia enterocolitica*
Veillonella atypica	*Yersinia intermedia*
Veillonella denticariosi	*Yersinia pseudotuberculosis*
Veillonella dispar	
Veillonella parvula	
Veillonella rodentium	
Veillonella rogosae	
Veillonella tobetsuensis	
Veillonella uc	
