# Supplementary material for: Changes in Gastric Corpus Microbiota With Age and After Helicobacter pylori Eradication: A Long-Term Follow-Up Study
Source: Front Microbiol. 2021 Feb 9;11:621879. doi: 10.3389/fmicb.2020.621879 (PMC7900007; doi:10.3389/fmicb.2020.621879)
Supplement: Supplementary file 16 [file Table_2.PDF]

Supplementary Table S2. Summary of Taxonmic Associations with Aging in Gastric Mucosa-Associated Microbiota at Corpus.

Phylum	Class	Order	Family	Genus
	**Alphaproteobacteria**	Sphingomonadales	Sphingomonadaceae	***Sphingobium***
**Proteobacteria**	Betaproteobacteria	**Burkholderiales**	**Comamonadaceae**	
	**Gammaproteobacteria**	**Pseudomonadales**	**Moraxellaceae**	***Enhydrobacter***
	**Bacilli**	**Lactobacillales**	**Streptococcaceae**	***Streptococcus***
**Firmicutes**	**Negativicutes**	**Veillonellales**		
	**Clostridia**	**Clostridiales**	**Veillonellaceae**	***Veillonella***
**Fusobacteria**	**Fusobacteriia**	**Fusobacteriales**	**Fusobacteriaceae**	***Fusobacterium***
			**Nocardioidaceae**	
Actinobacteria	Actinobacteria	**Actinomycetales**		
			**Actinomycetaceae**	***Rothia***
Bacteroidetes	**Bacteroidia**	**Bacteroidales**	**Prevotellaceae**	***Prevotella***
	Flavobacteriia	Flavobacteriales	Flavobacteraceae	***Chryseobacterium***

Red color indicates significant taxa of which relative abundance was increased with aging; blue color indicates significant taxa of which relative abundance was decreased with aging. **Bold** style indicates significant taxa (false discovery rate q-value < 0.05).
